# Fe_3_O_4_/PANI/CuI as a sustainable heterogeneous nanocatalyst for A^3^ coupling†

**DOI:** 10.1039/d4na00448e

**Published:** 2024-07-22

**Authors:** Nisha, Sahil Kohli, Snigdha Singh, Neera Sharma, Ramesh Chandra

**Affiliations:** a Drug Discovery & Development Laboratory, Department of Chemistry, University of Delhi Delhi-110007 India acbrdu@hotmail.com; b Department of Chemistry, School of Basic Sciences, Galgotias University Greater Noida-203201 Uttar Pradesh India; c Department of Chemistry, Hindu College, University of Delhi Delhi-110019 India; d Dr. B. R. Ambedkar Centre for Biomedical Research (ACBR), University of Delhi Delhi-110007 India; e Institute of Nanomedical Science (INMS), University of Delhi Delhi-110007 India; f Maharaja Surajmal Brij University Bharatpur-321201 Rajasthan India; g Manav Rachna International Institute of Research & Studies Faridabad Haryana-121004 India

## Abstract

The prepared copper iodide nanoparticles were impregnated on the support of ferrite nanoparticles functionalized with polyaniline, resulting in a magnetically recoverable heterogeneous nanocomposite. The activity of the prepared nanocomposite was investigated in the synthesis of propargylamine derivatives *via* A^3^ coupling under mild conditions. Techniques such as FESEM, EDAX, XRD, XPS, TEM, BET and FTIR were used to characterize the effective and unique heterogeneous Fe_3_O_4_/PANI/CuI nanocomposite developed in this work. This method used in the current study has several advantages, including a short reaction time, neat conditions, good product yield, ideal green matrices values, reusability for up to seven cycles, and magnetic retrievability.

## Abstract 2

The prepared copper iodide nanoparticles were impregnated on the support of ferrite nanoparticles functionalized with polyaniline, resulting in a magnetically recoverable heterogeneous nanocomposite.

## Graphical abstract

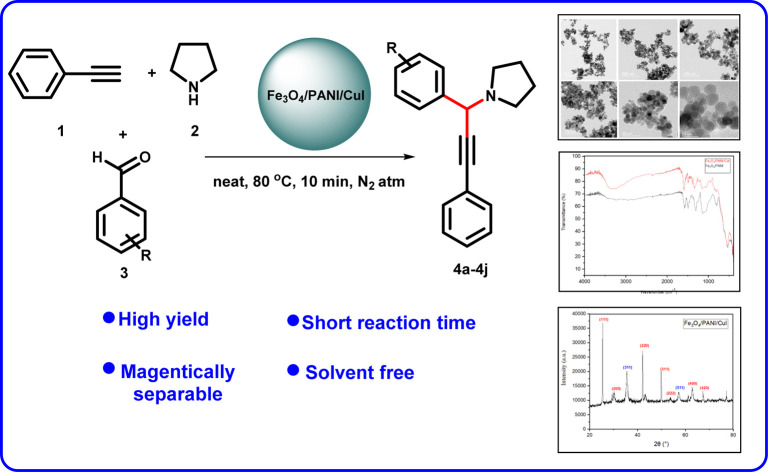

## Introduction

Magnetic nanoparticles (MNPs) are of great importance due to their various applications in catalysis, magnetic resonance imaging (MRI), magnetic fluids, and biotechnology.^1^ MNPs can serve as magnetically recoverable catalysts for a variety of catalytic reactions because of their insoluble and paramagnetic character, which allows for easy separation from the reaction medium. Moreover, magnetic separation has evolved into one of the most significant and well-known catalytic methods in organic chemistry without the need for filtering, centrifugation, or other laborious workup procedures, simply by using an external magnet.^2^ However, bare MNPs have some limitations such as the tendency to easily agglomerate, colloidal instability, and dissolution in acids.^3^ The colloidal instability of MNPs leads to their agglomeration due to magnetic dipole–dipole interaction.^4,5^ This issue can be resolved by surface functionalization of MNPs using protective shells or coatings such as silica, carbon, or organic polymers.^3^ Furthermore, these coatings allow the covalent attachment of organic compounds on distinct nanoparticles, facilitating applications such as drug carriers, heterogeneous catalysis, and absorption media.^3^ The magnetic nature of nanoparticles permits facile recovery of nanocatalysts from reaction mixtures through magnets.^5^

One of the polymeric shells synthesized *via* oxidative polymerization is polyaniline (PANI).^6^ The choice of polyaniline is because of its various properties such as facileness of synthesis, conductivity as a polymer, low cost, and a porous structure that can enhance the catalytic activity of nanoparticles. The Fe_3_O_4_/PANI hybrid shell can be considered a multifunctional support for metal nanocatalysts with significant catalytic performance.^7^

The benefits of a metal nanoparticle supported on nano-size heterogeneous material include good selectivity, minimal accumulation of metal nanoparticles, high dispersion in a liquid medium, and excellent reusability.^8^ Copper-based nanoparticles not only enhance the physicochemical characteristics of the nanoparticles but also reinforce the interface between the metal and the support.^9^ Cu-based nanocatalysts have abundant applications in nanotechnology due to their special properties and features such as catalysing organic transformations, electrocatalysis, and photocatalysis.^10^ Supported copper nanoparticles, such as CuO/NiO,^9^ CuO/Al_2_O_3_,^11^ ZnO/CuI/PPy,^12^ and Cu–MgO,^13^ have been used in many organic transformations. Copper-based nanocatalysts are found to be useful in various reactions including C–H activation of alkynes, oxygen arylation reaction, Suzuki reaction, Click reaction, Knoevenagel condensation-Michael addition cyclization reaction, Heck reaction and producing copper-acetylated species *in situ* to afford propargylamines.^14^

**Fig. 1:**
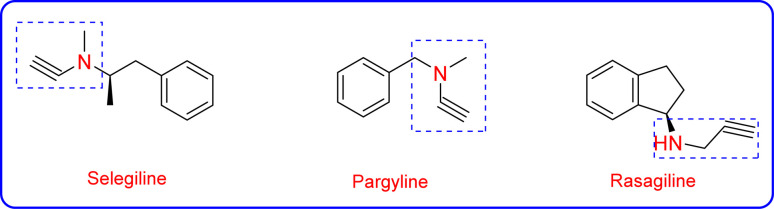
Examples of approved drugs containing propargylamine skeletons.

Propargylamines are crucial building blocks for organic synthesis because they can be utilized as synthetic precursors for synthesizing various medicinally essential compounds.^8^ Propargylamines are formed *via* a three-component reaction known as A^3^ coupling, which comprises a terminal alkyne, an aldehyde, and an amine.^15^ Moreover, a variety of propargylamines have been used to cure neuropsychiatric conditions like anxiety, Parkinson's disease, and depression.^16^ Various approved drugs, such as pargyline, selegiline, and rasagiline (Fig. 1), have a propargylamine scaffold.^8,17^ Late transition metals such as Au, Cu, and Ag are used to catalyse A^3^ reactions *via* one-pot synthesis.^18^

Over the past two decades, a catalytic variation of A^3^ coupling has attracted chemists' attention^15^ A^3^ coupling catalysed by various nanocatalysts such as Fe_3_O_4_@*R. tinctorum*/Ag,^19^ Cu/C,^16^ Fe_3_O_4_@SiO_2_@DNHCS-Tr@CuI,^20^ Au nanoparticles,^21^ Fe_3_O_4_–MoO_3_ (ref. 22) and CuO/GNS^23^ have been reported. However, these methods involve use of harmful reagents, prolonged reaction time, use of additives and costly reagents. Hence, there is a need for a sustainable heterogeneous nanocatalyst for the facile synthesis of propargylamines *via* A^3^ coupling.

In the current work, we successfully develop a novel heterogeneous nanocatalyst, Fe_3_O_4_/PANI/CuI, for the synthesis of propargylamines *via* A^3^ coupling using pyrrolidine, phenylacetylene, and different benzaldehydes under neat conditions at 80 °C in a N_2_ atmosphere. The reaction was completed in 10 min with a high yield of the desired product. The fabricated nanocatalyst was easily recoverable and reusable with high catalytic efficiency for the synthesis of propargylamines.

## Results and discussion

### Synthesis and characterisation

#### Synthesis of Fe_3_O_4_ nanoparticles

Fe_3_O_4_ nanoparticles were synthesised using the co-precipitation approach. To a 250 mL round bottom flask containing 100 mL of water, 4.2 g of FeSO_4_·7H_2_O and 6.1 g of FeCl_3_·6H_2_O were added. The mixture was stirred at 80 °C for 1 h. Then, 10 mL of ammonia solution (25%) was added dropwise into the reaction mixture with continuous stirring. Then, the reaction was continuously stirred for another 1.5 h at the same temperature. The Fe_3_O_4_ nanoparticles were collected using a magnet and washed with water many times and then with ethanol. Finally, the synthesised nanoparticles were dried in an oven at 50 °C.^5^

#### Synthesis of Fe_3_O_4_/PANI nanoparticles

The obtained Fe_3_O_4_ nanoparticles were dispersed in 10 mL of deionized water. Subsequently, 0.3 mL of HCL (0.1 M) and 0.2 mL of aniline were added to this solution. Then, the solution was stirred for 1 h at room temperature. Then, the aqueous solution of ammonium persulfate (5 mL) was poured dropwise to the above reaction under ultrasonic irradiation. The stirring was then continued for 3 h in an ice bath. Then, nanoparticles were collected with a magnet and then washed many times with water and three times with ethanol, and further dried in an oven at 50 °C.

#### Synthesis of Fe_3_O_4_/PANI/CuI nanoparticles

**Fig. 2:**
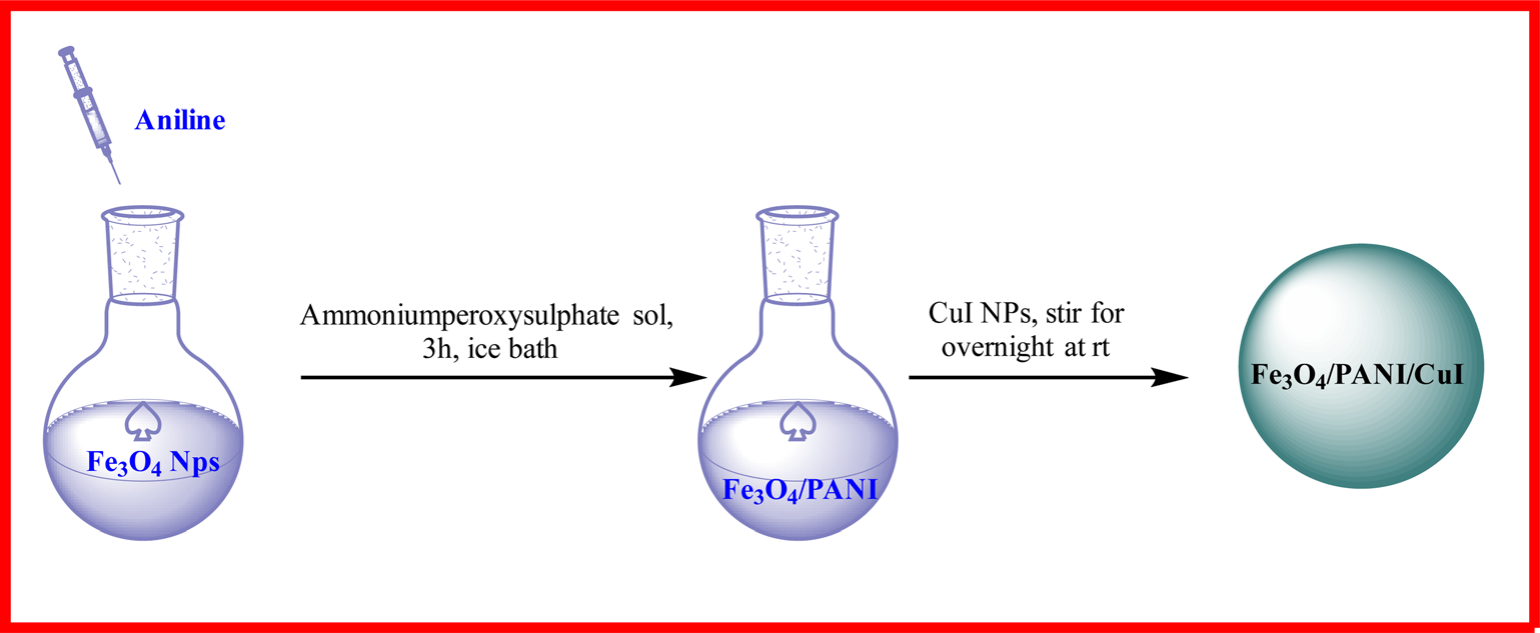
Schematic diagram for the synthesis of nanocomposite.

The prepared Fe_3_O_4_/PANI nanoparticles were dispersed in water *via* stirring for 10 min. Then, copper iodide nanoparticles were added to this solution and it was stirred overnight at room temperature. Then, nanoparticles were washed with water and ethanol and dried in an oven overnight at 50 °C (Fig. 2).

#### Characterisation of developed Fe_3_O_4_/PANI/CuI nanocatalyst

**Fig. 3:**
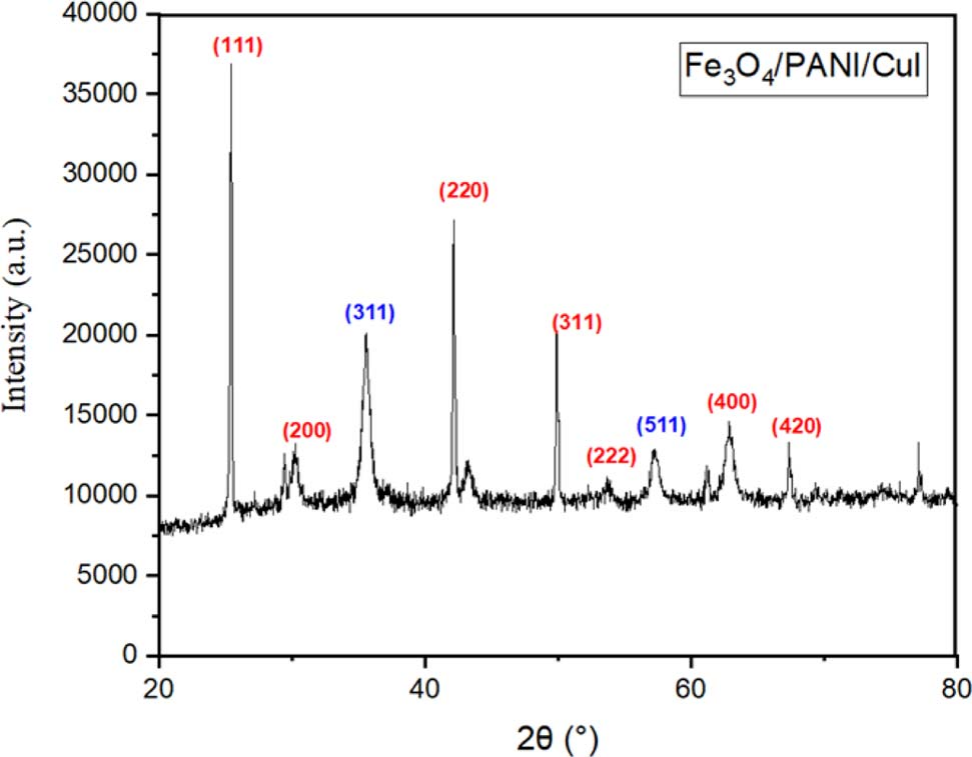
XRD pattern of Fe_3_O_4_/PANI/CuI nanocatalyst.

X-ray diffraction analysis of the Fe_3_O_4_/PANI/CuI nanocomposite is shown in Fig. 3. The diffraction angles (2*θ*) at 35.46° and 57.06° correspond to the crystal planes (311) and (511), respectively, of the Fe_3_O_4_ nanoparticles.^24,25^ The peaks at 2*θ* = 25.42°, 30.06°, 42.2°, 49.86°, 57.14°, 61.22°, and 67.3° correspond to the crystal planes (200), (311), (111), (420), (222), (220), and (420), respectively, of the cubic phase of CuI.^26^

**Fig. 4:**
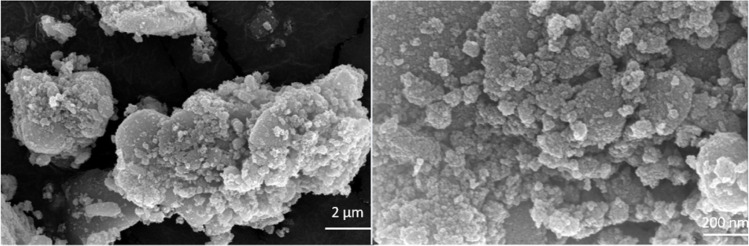
FESEM analysis of Fe_3_O_4_/PANI/CuI nanocatalyst.

**Fig. 5:**
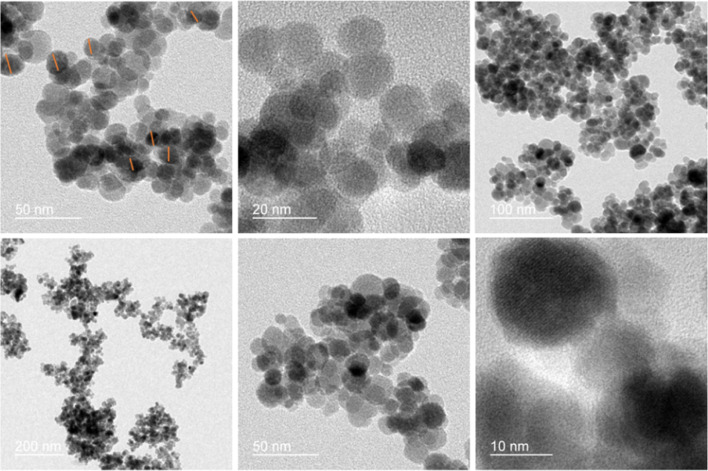
TEM analysis of Fe_3_O_4_/PANI/CuI nanocatalyst.

The field-emission scanning electron microscopy (FESEM) technique reveals the spherical morphology of the nanocomposite (Fig. 4). The transmission electron microscopy (TEM) analysis of the Fe_3_O_4_/PANI/CuI nanocomposite indicates that CuI is well embedded over the core–shell structure of Fe_3_O_4_ nanoparticles and the average size of nanoparticles is 42.6 nm, as shown in Fig. 5.

**Fig. 6:**
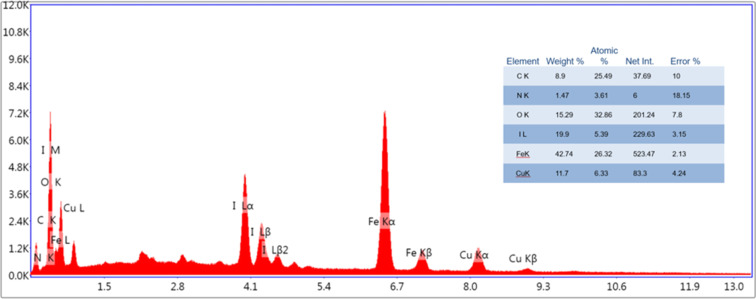
EDAX analysis of Fe_3_O_4_/PANI/CuI nanocatalyst.

The energy-dispersive X-ray analysis of the Fe_3_O_4_/PANI/CuI nanocatalyst revealed the presence of iron (42.74 wt%), nitrogen (1.47 wt%), copper (11.7 wt%), oxygen (15.29 wt%), iodine (19.9 wt%), and carbon (8.9 wt%), as can be seen in Fig. 6.

**Fig. 7:**
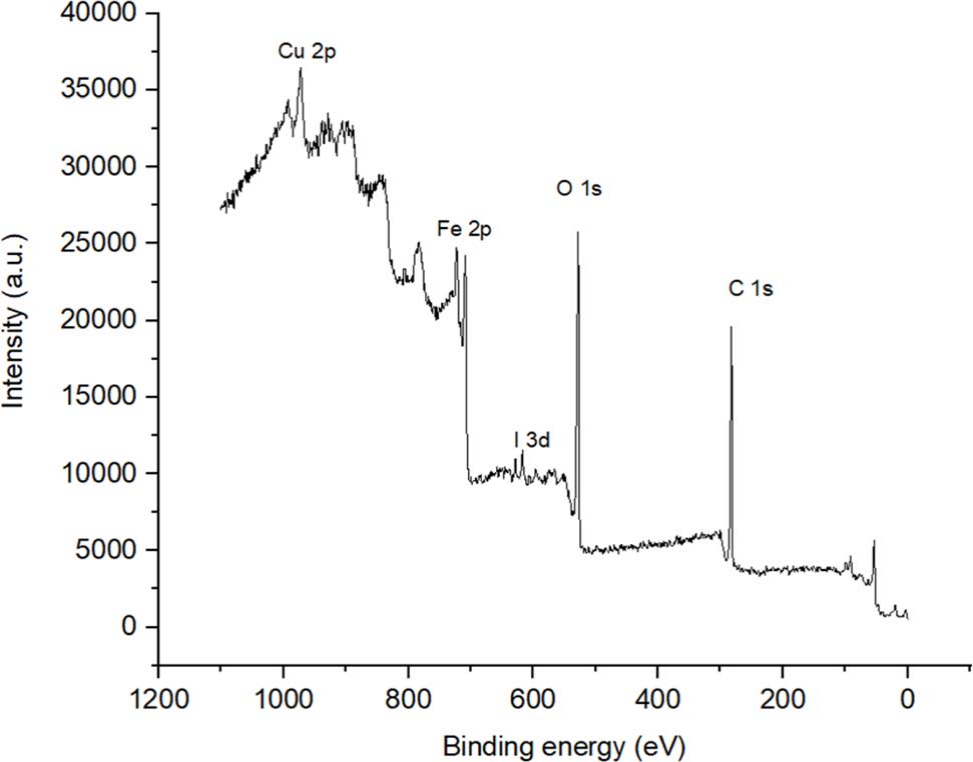
XPS of Fe_3_O_4_/PANI/CuI nanocatalyst.

Fig. 7 illustrates the X-ray photoelectron spectra (XPS) of the Fe_3_O_4_/PANI/CuI nanocomposite. The spectra revealed the presence of Cu 2p_1/2_ and Cu 2p_3/2_ with binding energies at 952.88 and 932.44 eV, respectively, and the presence of I 3d_3/2_ and 3d_5/2_ with binding energies at 631 and 619.23 eV, respectively. The values for copper and iodine resemble the reported binding energy values of CuI, which confirm the +1 oxidation state of copper in the nanocomposite.^27^ The peak at 284.78 eV corresponds to the binding energy value of C 1s. The values of binding energies at 724.53 and 710.63 eV resemble the reported values of Fe 2p_3/2_ and Fe 2p_1/2_, respectively, while the peak at 530.17 eV corresponds to O 1s, confirming the presence of Fe_3_O_4_ in the nanocomposite. The broadness of the iron peaks indicates the presence of both oxidation states (Fe^2+^ and Fe^3+^) in Fe_3_O_4_.^28^

**Fig. 8:**
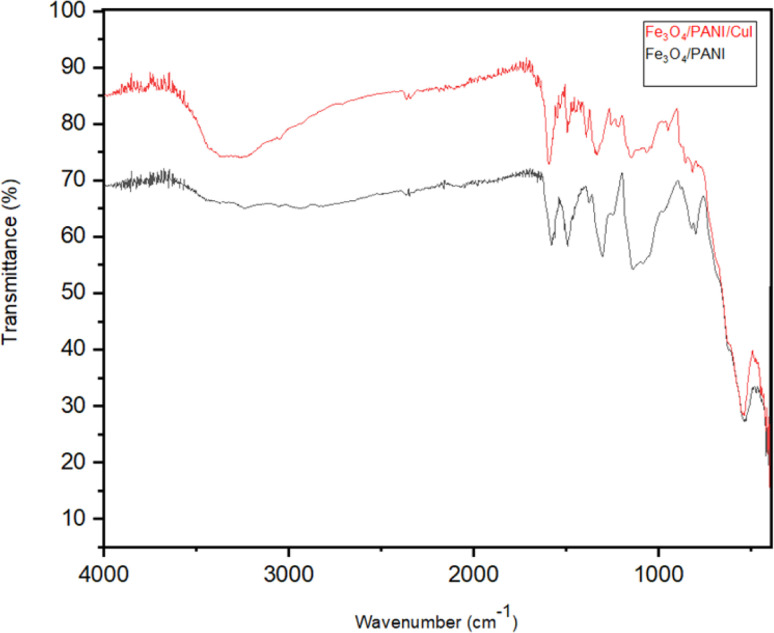
FTIR of Fe_3_O_4_/PANI/CuI nanocatalyst.

Fig. 8 shows the FTIR spectrum of Fe_3_O_4_/PANI/CuI; it depicts a peak at 3311 cm^−1^, which is attributed to the presence of the surface OH group in the nanocomposite.^29^ The peaks at 1598 and 1494 cm^−1^ are attributed to the C=C stretching vibrations of a quinoid and benzenoid ring, respectively.^30^ A peak that appeared at 1374 cm^−1^ is similarly typical of polyaniline and is considered to be a consequence of C–N stretching vibrations near a quinonoid ring.^31^ The peak at 1161 cm^−1^ is due to the C–N stretching vibration.^30^ The peak at 553 cm^−1^ is the characteristic peak of ferrite nanoparticles.^30^

**Fig. 9:**
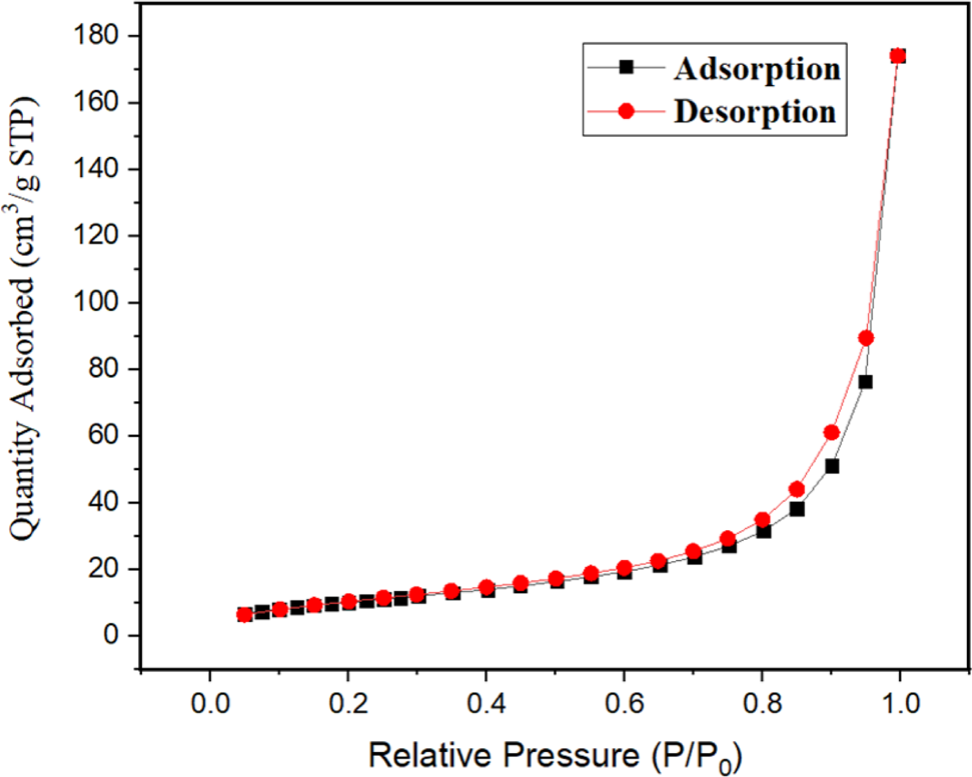
N_2_ adsorption–desorption isotherm of nanocatalyst.

N_2_-Adsorption desorption isotherm was collected using the Brunauer–Emmett–Teller (BET) technique, which is portrayed as a H3 hysteresis loop of isotherm and shows a surface area of 38.471 m^2^ g^−1^, pore radius of 2.16 nm, and pore volume of 0.076 cm^3^ g^−1^ (Fig. 9).

### Fe_3_O_4_/PANI/CuI as heterogeneous nanocatalysts for the synthesis of propargylamine derivatives

**Scheme 1:**
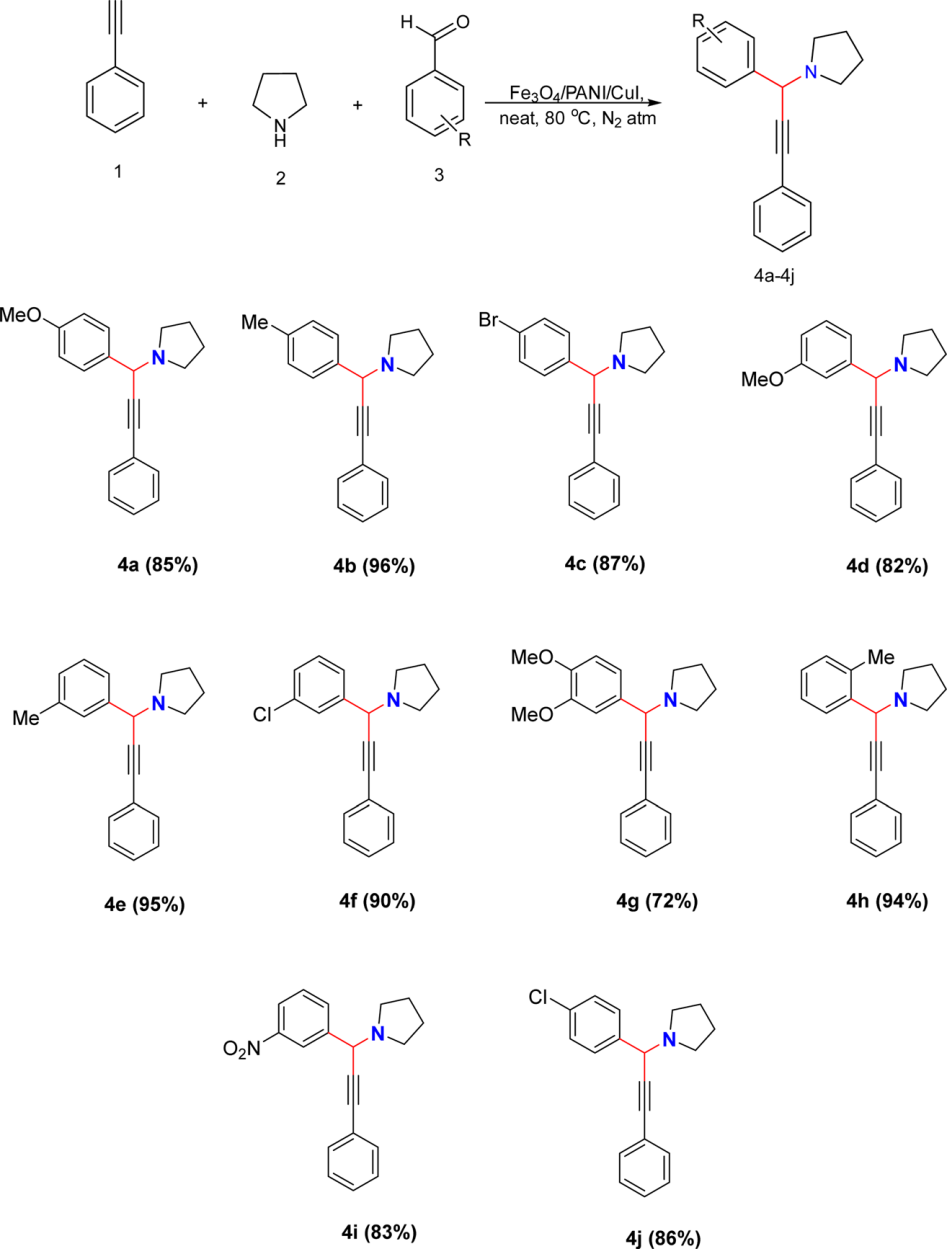
Fe_3_O_4_/PANI/CuI catalysed synthesis of propargyl derivatives *via* A^3^ coupling. Reaction conditions: nanocatalyst (10 mg), phenylacetylene (1 mmol), pyrrolidine (1 mmol), aromatic aldehyde (1 mmol), neat, 80 °C, N_2_ atm, 10 min.

We synthesized propargylamine derivatives using Fe_3_O_4_/PANI/CuI to investigate its catalytic properties in organic transformations (Scheme 1). For optimization, a model reaction was performed involving phenylacetylene (1), pyrrolidine (2), and 4-methyl benzaldehyde (3) using the nanocatalyst in various solvents or under neat conditions at 80 °C in a nitrogen atmosphere for the preparation of the desired product 4b, as shown in Table 1. We examined how different catalyst loading amounts, solvent concentrations, and temperatures affected the reaction kinetics, as presented in Table 1. Initially, the model reaction was performed in toluene (Table 1, entry 1), resulting in a 47% yield of the product. Subsequently, the reaction was carried out in polar aprotic solvents such as THF, acetonitrile, DMSO, and DMF. The desired product did not form in both acetonitrile and THF (Table 1, entries 2 and 3). However, the product was obtained with a 40% yield in DMF (Table 1, entry 4). The reaction was then monitored in environmentally friendly solvents such as ethanol, water, and ethylene glycol (EG), yielding no product in water (Table 1, entry 5), trace amounts of product in ethanol, and 30% product yield in EG (Table 1, entry 7). The product was isolated in good yield in neat conditions (Table 1, entry 8). By altering the catalyst loading, the % yield was found to remain unchanged on lowering or increasing the catalyst amount respectively (Table 1, entries 9 and 10). Further, we studied the influence of temperature on development of reaction. On raising the temperature, there was no change in product yield (Table 1 entry 11), while on decreasing the temperature, there was a reduction in the product yield (Table 1, entry 12).

**Table 1:** Optimization of nanocatalyst for the synthesis of propargyl derivatives *via* A^3^ coupling using phenyl acetylene (1), pyrrolidine (2), and 4-methylbenzaldehyde (3)^a^

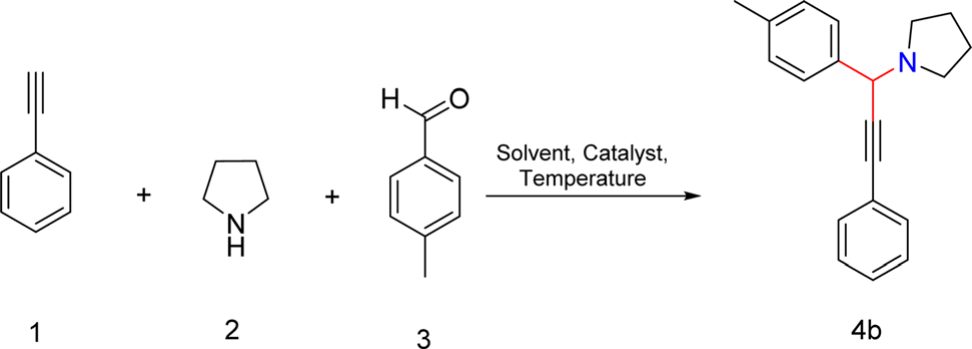					
S. no.	Nanocatalyst (mg)	Solvent	Temp. (°C)	Time (min)	Yield (%)
1	Fe_3_O_4_/PANI/CuI (10)	Toluene	80	10	47
2	Fe_3_O_4_/PANI/CuI (10)	CH_3_CN	80	10	—
3	Fe_3_O_4_/PANI/CuI (10)	THF	80	10	—
4	Fe_3_O_4_/PANI/CuI (10)	DMF	80	10	40
5	Fe_3_O_4_/PANI/CuI (10)	Water	80	10	—
6	Fe_3_O_4_/PANI/CuI (10)	Ethanol	80	10	Trace
7	Fe_3_O_4_/PANI/CuI (10)	EG	80	10	30
**8**	**Fe_3_O_4_/PANI/CuI (10)**	**Neat**	**80**	**10**	**96**
9	Fe_3_O_4_/PANI/CuI (5)	Neat	80	10	53
10	Fe_3_O_4_/PANI/CuI (20)	Neat	80	10	96
11	Fe_3_O_4_/PANI/CuI (10)	Neat	110	10	96
12	Fe_3_O_4_/PANI/CuI (10)	Neat	50	10	22
13	CuI (10)	Neat	80	10	41

a Reaction conditions: catalyst (5–20 mg), 1 (1.0 mmol), 2 (1 mmol), 3 (1 mmol), solvent (2–3 mL), N_2_ atm, 80 °C, 10 min.

**Fig. 10:**
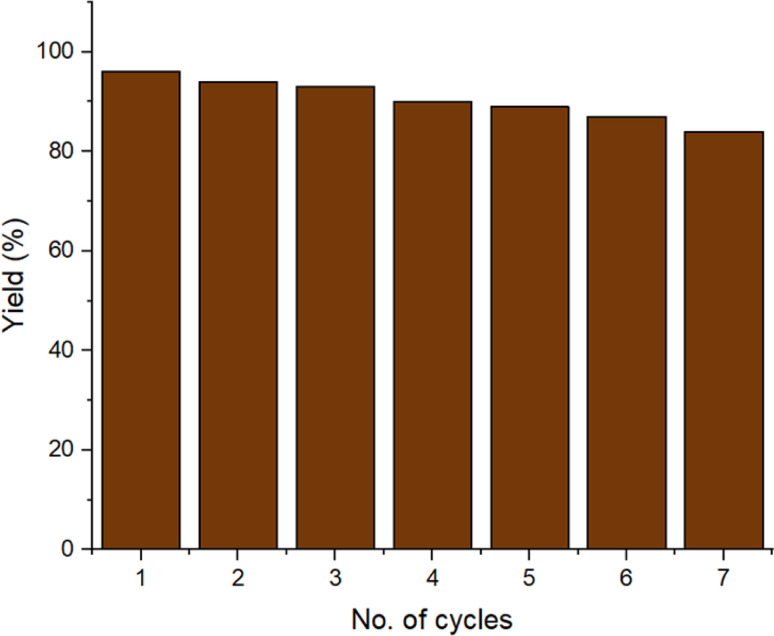
Catalyst recyclability test.

Under optimized conditions, we examined the recyclability of the catalyst to produce the product 4b. Once the reaction was completed, the catalyst was recovered from the reaction using a magnet and then washed many times with water and ethanol before being dried in the oven. The recovered catalyst was then used for seven cycles (Fig. 10). The stability of the recycled catalyst after seven cycles was confirmed by XRD, SEM, FTIR, EDAX and TEM, which confirmed that there was no change in the activity and morphology of the catalyst (ESI Fig. S1–S5†). An ICP study of the filtrate was done after catalyst recovery and showed the leached metal concentrations of copper and iron ion to be 2.08 and 0.12 ppm, respectively, which are lower than the authentic values of the respective ions according to WHO terms.^32^

The existing methodology demonstrates sustainability and eco-friendliness, as evidenced by the green metrics values, as shown in Table 2 (refer to calculations in the ESI†), which closely approach the ideal values.

**Table 2:** Green matrices values

Catalyst	Reaction mass efficiency	*E*-Factor	Process mass intensity	Carbon efficiency
Fe_3_O_4_/PANI/CuI	90%	0.10	1.10	96%

Table 3 provides a summary of the literature review, listing the previously established methods for producing propargyl derivatives, including the reaction conditions and corresponding yields.

**Table 3:** Comparative analysis of various catalysts for the synthesis of propargylamine derivatives

S. no.	Nanocatalyst	Reaction conditions	Time	% Yield	Ref.
1	Fe_3_O_4_@SiO_2_–Se-T/CuI	Neat, 80 °C	2 h	95	33
2	ZSM-5/APTMS/(*E*)-4-((pyridine-2-ylimino)methyl)benzaldehyde/Cu-NPs	K_2_CO_3_, H_2_O, 60 °C	2 h	94	34
3	UIO-66-NH_2_G1@PdNPs	Toluene, N_2_ gas, 110 °C	3 h	93	35
4	[Fe_3_O_4_@bisimidazolium-Pd]^2Cl^−^^	PEG-400, 100 °C	2 h	98	36
5	Fe_3_O_4_@starch-Acr@Cu(ii)	H_2_O, reflux	35 min	99	37
6	g-C_3_N_4_-TCT-2AEDSEA-Ag-Cu-Ni	Toluene, 80 °C	8 h	91	38
7	Fe_3_O_4_@SiO_2_-di-(pyridin-2-yl)amine-Cu	H_2_O, reflux	2 h	99	39
8	Co^2+^-Cu@SA(0)-600	Toluene, 110 °C	1 h	89	40
9	MMT-K10/Fe_3_O_4_/CuO	Toluene, 80 °C	8 h	91	41
10	*o*-Cu_2_O-PVP	Neat, 100 °C	5 min	80	42
**11**	**Fe_3_O_4_/PANI/CuI**	**Neat, 80 °C**	**10 min**	**96**	**Our work**

**Fig. 11:**
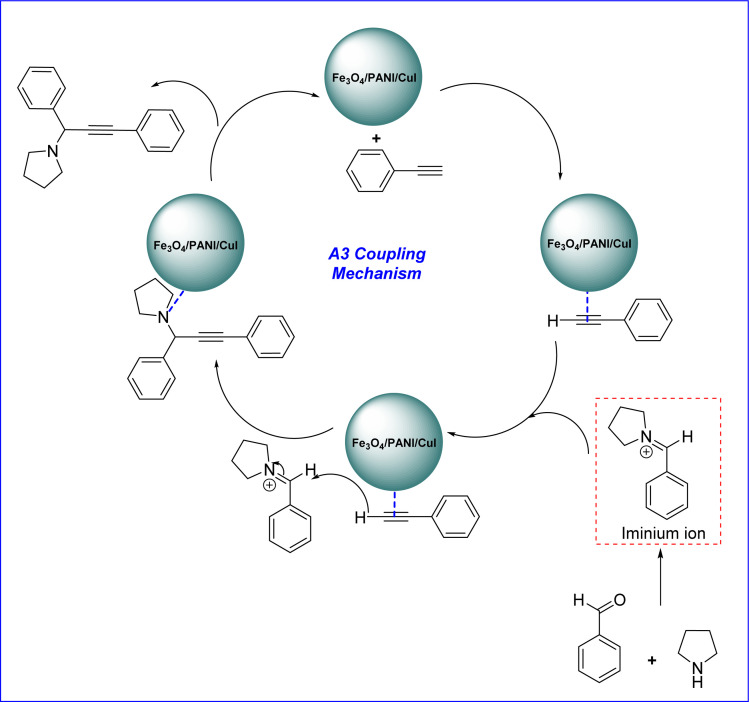
Mechanism for Fe_3_O_4_/PANI/CuI catalysed synthesis of propargylamine derivative *via* A^3^ coupling.

The plausible mechanism for the synthesis of propargylamine *via* A^3^ coupling catalysed by the Fe_3_O_4_/PANI/CuI nanocomposite is shown in Fig. 11. The copper-based nanocatalyst activates the phenylacetylene ring and proceeds through an attack on the carbon of the iminium ion, which is formed from the aldehyde and amine and results in the formation of the desired product as well as catalyst regeneration.^33,39^

### General procedure for the synthesis of propargyl derivatives

In general, a mixture of phenylacetylene (1 mmol), pyrrolidine (1 mmol), aromatic aldehyde (1 mmol), and catalyst (10 mg) was added to a 50 mL round-bottom flask and stirred continuously at 80 °C. TLC was used to monitor the progress of the reaction. After the completion of the reaction, the reaction mixture was cooled and diluted with ethyl acetate, and the catalyst was separated with the aid of a magnet. The crude product was extracted with ethyl acetate and purified by column chromatography using basic alumina as a stationary phase and ethyl acetate : hexane as an eluent. The obtained pure product was confirmed by ^1^H and ^13^C NMR spectroscopy.

## Conclusion

In summary, we have developed a sustainable heterogeneous copper-based magnetic nanocatalyst for the one-pot synthesis of propargylamine derivatives under solvent-free conditions with a short reaction time. The designed nanocatalyst is easily magnetically recoverable and can be recycled for up to seven runs without any drastic reduction in product yield. This protocol provides a shorter reaction time to obtain products with high yield and good catalytic activity under mild reaction conditions as compared to previously reported methods.

## Data availability

The data that support the findings of this study are available from the corresponding author following reasonable request.

## Conflicts of interest

The authors declare no conflicts of interest.

## Supplementary Material

NA-006-D4NA00448E-s001
