# Supplementary material for: Fe3O4/PANI/CuI as a sustainable heterogeneous nanocatalyst for A3 coupling
Source: Nanoscale Adv. 2024 Jul 22;6(19):4842–51. doi: 10.1039/d4na00448e (PMC11421548; doi:10.1039/d4na00448e)
Supplement: NA-006-D4NA00448E-s001 [file NA-006-D4NA00448E-s001.pdf]

Electronic Supplementary Material (ESI) for Nanoscale Advances.
This journal is © The Royal Society of Chemistry 2024

Supporting Information

Fe_3O_4/PANI/CuI as sustainable heterogenous nanocatalyst for A^3 coupling

Nisha[a], Sahil Kohli[a,f], Snigdha Singh,[a] Neera Sharma[b*], Ramesh Chandra[a,c,d*]

[a] Drug Discovery & Development Laboratory, Department of Chemistry, University of Delhi, Delhi-110007, India.

[b] Department of Chemistry, Hindu College, University of Delhi, Delhi-110019, India.

[c] Dr. B. R. Ambedkar Centre for Biomedical Research (ACBR), University of Delhi, Delhi-110007, India.

[d] Institute of Nanomedical Science (INMS), University of Delhi, Delhi-110007, India.

[e] Maharaja Surajmal Brij University, Bharatpur 321201, Rajasthan, India.

[f] Department of chemistry, school of basic sciences, Galgotias University, Greater Noida-203201, Uttar Pradesh India.

Electronic supplementary information (ESI) includes FESEM, XRD, FTIR and TEM of recycled Fe_3O_4/PANI/CuI nanocomposite; green chemistry matric calculation, ^{1}H NMR and ^{13}C NMR of compounds.

Total no. of Pages: 26, Total no. of Figures: 24

Table of contents

Serial number	Content	Page number
1.	General Remarks	2
2.	FESEM, XRD, TEM and FTIR of recycled Fe_3O_4/PANI/CuI nanocatalyst	2-4
4.	Calculation of green chemistry matrices	4-5
5.	Spectral data of compounds	6-7
6.	^{1}H and ^{13}C spectra of compounds	8-26

1. **General Remark**

Chemicals and solvents were purchased from sigma aldrich, alfa-aesar and Merck India pvt.^{1}H and ^{13}C spectra were recorded on a Jeol Spectrospin spectrometer at 400 MHz and 100 MHz respectively by keeping TMS as internal standard. Chemical shift values were recorded in terms of δ and coupling constant in hertz (Hz). The X-ray diffractometer (Model No. D8 DISCOVER) at 2θ range of 5–90° with Cu Kα radiation. FTIR spectra were obtained on IR affinity 1S- Fourier Transform Infrared spectrophotometer. Scanning electron microscopy was performed on JEOL JSM 6610 at USIC, University of Delhi. Transmission electron microscopy was obtained on a TECNAI 200 HR-TEM. The elemental composition and electronic structure analysis were obtained from X-ray photoelectron spectra (XPS) of Thermofisher- company model-K@.

2. FESEM, XRD, TEM and FTIR (Figure S1 S2 S3 and S4) of Recycled Nanocatalyst

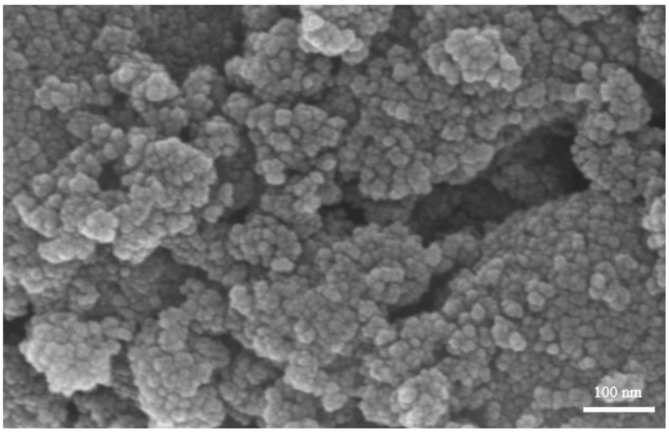

Figure S1: FESEM image of recycled nanocatalyst

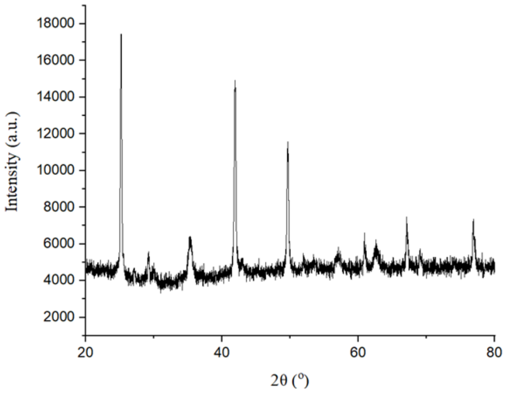

Figure S2: XRD of recycled nanocatalyst

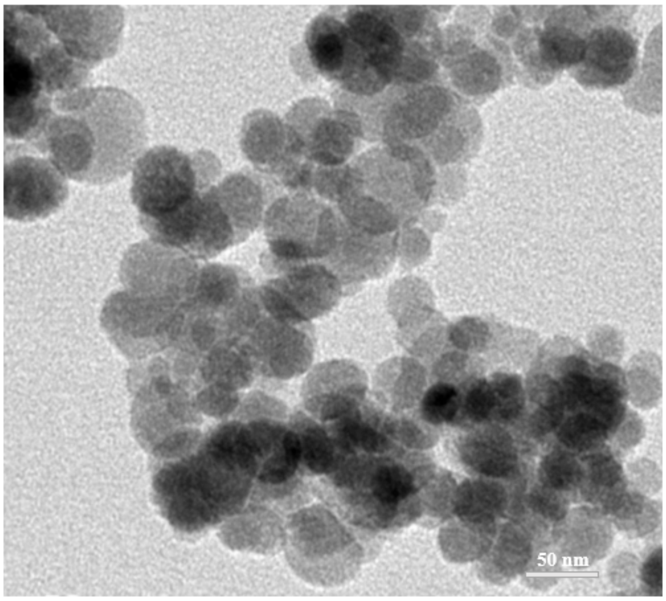

Figure S3: TEM image of recycled nanocatalyst

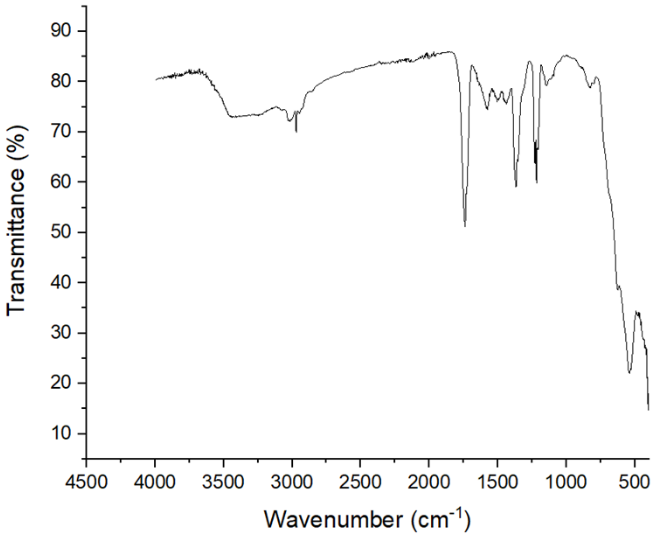

Figure S4: FTIR of recycled nanocatalyst

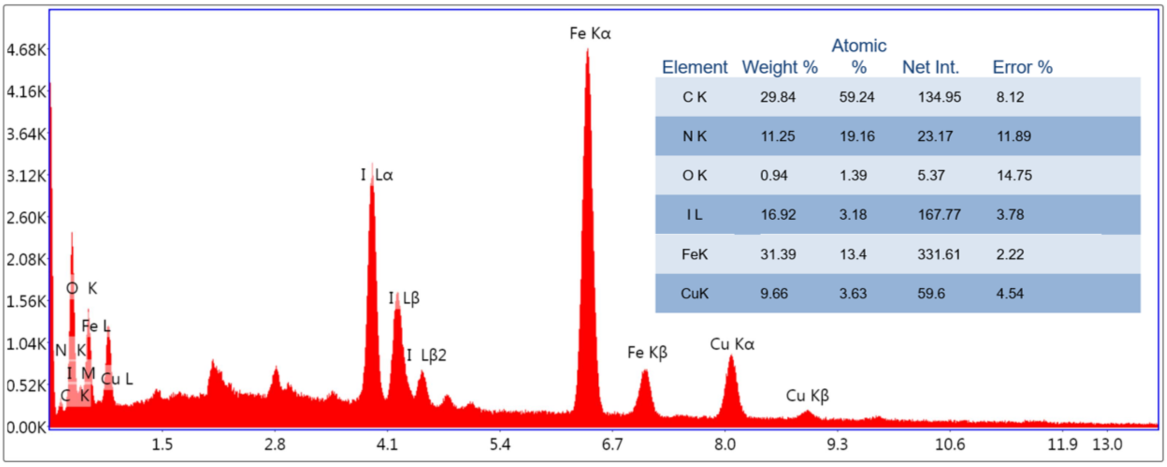

Figure S5: EDAX of recycled nanocatalyst

3. Calculation of Green Chemistry Matrices for 4b

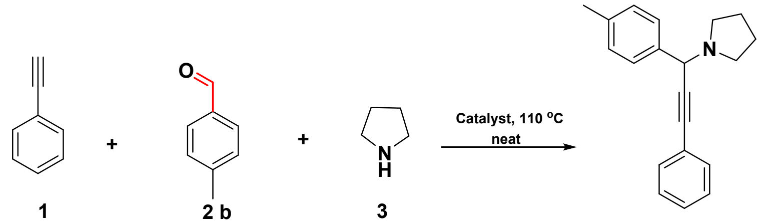

1 2 b 3 4b

	1	2 b	3	4b
FW	102.14 g/mol	120.15 g/mol	71.12 g/mol	275.40 g/mol
mmol	1.0	1.0	1.0	0.96
amount	102 mg	120 mg	71 mg	264 mg

E-factor:

The ideal value of E-factor is zero.

E-factor = [total mass of raw materials-total mass of product]/mass of product.

E-factor of 4b = [(102+120+71) - 264]/264

= 0.10

Process Mass Intensity (PMI):

PMI = ∑ (mass of stoichiometric reactants)/ [mass of product]

= (102+120+71)/ 264

= 1.10

Reaction Mass Efficiency (RME):

RME = [mass of product /Σ (mass of stoichiometric reactants)] x 100

= [264 / (102+120+71)] x 100

= 90 %

Carbon Efficiency (CE):

CE denotes the percentage of carbon in the reactants that remains in the product.

CE = [Amount of carbon in product/ Total carbon present in reactants] x 100

= [no. of moles of product x no. of carbons in product / (no. of moles of 1 x no. of carbons in 1 + no. of moles of 2 x no. of carbons in 2 + no. of moles of 3 x no. of carbon in 3)] x 100

= [(0.96 x 20) / (1.0 x 8 + 1.0 x 8 + 1.0 x 4)] x 100

= [19.2/ (8 + 8 + 4)] x 100

= 96 %

4. Spectral data of compounds

1-(1-(4-methoxyphenyl)-3-phenylprop-2-yn-1-yl)pyrrolidine[4a]

Solid; Yield: 85%; ^{1}H NMR ($CDCl_3$, 400 MHz): δ 7.52-7.50 (d, *J* = 8.7 Hz, 2H), 7.48 (s, 1H),7.31-7.29 (m, 4H) 6.89-6.87 (d, *J* = 8.7 Hz, 2H), 4.82 (s, 1H), 3.80 (s, 3H) 2.67 (t, *J* = 6.7 Hz, 4H), 1.79 (t, *J* = 6.4 Hz, 4H). ^{13}C NMR ($CDCl_3$, 400 MHz): δ 159.11, 131.89, 131.86, 131.83, 131.77, 129.49, 128.37, 128.34, 128.31, 128.14, 123.34, 113.69, 113.66, 113.63, 87.07, 56.74, 58.60, 55.38, 50.35, 50.32, 23.53

1-(3-phenyl-1-(*p*-tolyl) prop-2-yn-1-yl)pyrrolidine[4b]

Solid; Yield: 96%; ^{1}H NMR ($CDCl_3$, 400 MHz): δ NMR (400 MHz,) δ 7.51-7.48 (m, 4H), 7.32-7.31 (m, 4H), 7.18 (d, *J* = 8.0 Hz, 2H), 4.87 (s, 1H), 2.73-2.69 (t, *J* = 6.7 Hz, 4H), 2.36 (s, 3H), 1.83-1.79 (t, *J* = 6.3 Hz, 4H).^{13}C NMR ($CDCl_3$, 400 MHz): δ 137.38, 136.50, 131.89, 129.10, 129.07, 129.04, 128.41, 128.39, 128.36, 128.33, 123.37, 87.00, 86.80, 58.97, 50.42, 50.39, 23.56, 21.25

1-(1-(4-bromophenyl)-3-phenylprop-2-yn-1-yl)pyrrolidine[4c]

Solid; Yield: 87%; ^{1}H NMR ($CDCl_3$, 400 MHz): δ 7.52-7.47 (m, 6H), 7.33-7.32 (m, 3H), 4.87 (s, 1H), 2.69-2.57 (t, J = 22.3 Hz, 4H), 1.80-1.66 (m, 4H) ^{13}C NMR ($CDCl_3$, 400 MHz): δ 138.69, 131.91, 131.46, 130.08, 128.43, 128.38, 123.04, 121.59, 87.48, 85.98, 58.50, 50.23, 23.65, 23.63

1-(1-(3-methoxyphenyl)-3-phenylprop-2-yn-1-yl)pyrrolidine[4d]

Solid; Yield: 82%; ^{1}H NMR ($CDCl_3$, 400 MHz): δ ^{13}C NMR ($CDCl_3$, 400 MHz): δ 7.53-7.51 (m, 2H), 7.34-7.28 (m, 4H), 7.25-7.23 (d, J = 7.1 Hz, 2H), 6.88 (d, J = 1.4 Hz, 1H), 4.88 (s, 1H), 3.84 (s, 3H), 2.76-2.72 (m, 4H), 1.85-1.82 (m, 4H) ^{13}C NMR ($CDCl_3$, 400 MHz): δ 159.76,

141.28, 131.92, 129.34, 128.41, 128.23, 123.35, 120.80, 120.80, 114.02, 113.17, 86.99, 86.85, 59.27, 55.35, 50.52, 50.49, 50.46, 23.66, 23.63

1-(3-phenyl-1-(*m*-tolyl) prop-2-yn-1-yl)pyrrolidine[4e]

Solid; Yield: 95%; ^{1}H NMR ($CDCl_3$, 400 MHz): δ ^{13}C NMR ($CDCl_3$, 400 MHz): δ 7.55-7.53 (m, 2H), 7.47-7.45 (m, 2H), 7.36-7.32 (m, 3H), 7.30-7.27 (m, 1H), 7.16-7.14 (dd, J = 9.3, 4.1Hz, 1H), 4.88 (s, 1H), 2.76-2.74 (m, 4H), 2.42 (s, 3H), 1.87-1.83 (t, J = 6.5 Hz, 4H) ^{13}C NMR ($CDCl_3$, 400 MHz): δ 139.53, 138.04, 131.93, 128.52, 128.40, 128.29, 128.20, 125.52, 123.43, 87.05, 86.89, 59.37, 50.57, 50.55, 50.52, 23.66, 23.63, 23.60, 21.64

1-(1-(3-chlorophenyl)-3-phenylprop-2-yn-1-yl)pyrrolidine[4f]

Solid; Yield: 90%; ^{1}H NMR ($CDCl_3$, 400 MHz): δ 7.65 (d, J = 1.8Hz, 1H), 7.53-7.50 (m, 3H), 7.34-7.31 (m, 3H), 7.30-7.28 (m, 2H), 7.25 (s, 1H), 4.91 (s, 1H), 2.72-2.68 (m, 4H), 1.83-1.80 (m, 4H), ^{13}C NMR ($CDCl_3$, 400 MHz): δ 141.77, 134.28, 131.95, 131.90, 129.63, 128.44, 128.40, 127.87, 126.52, 123.04, 58.60, 50.24, 23.65

1-(1-(3,4-methoxyphenyl)-3-phenylprop-2-yn-1-yl)pyrrolidine[4g]

Solid; Yield: 72%; ^{1}H NMR ($CDCl_3$, 400 MHz): δ 7.48-7.45 (m, 2H), 7.30-7.28 (m, 4H), 7.17-7.12 (m, 2H), 6.83 (d, J = 8.2 Hz 1H), 4.80 (s, 1H), 3.89 (s, 3H), 3.86 (s, 3H), 2.69 (t, J = 10.6, 6.8Hz, 4H), 1.80 (t, J = 5.4Hz, 4H), ^{13}C NMR ($CDCl_3$, 400 MHz): δ 148.91, 148.58, 131.83, 128.37, 128.21, 120.51, 111.50,110.70, 87.00, 86.84, 59.09, 56.03, 55.97, 50.52, 23.59

1-(3-phenyl-1-(*o*-tolyl) prop-2-yn-1-yl)pyrrolidine[4h]

Solid; Yield: 94%; ^{1}H NMR ($CDCl_3$, 400 MHz): δ 7.79-7.77 (m, 1H), 7.57-7.55 (m, 2H), 7.37-7.35 (m, 3H), 7.28-7.24 (m, 3H), 5.09 (s, 1H), 2.79 (t, J = 5.2 Hz, 4H), 2.54 (s, 3H), 1.85-1.82 (t, J = 6.2Hz , 4H), ^{13}C NMR ($CDCl_3$, 400 MHz): δ 137.96, 136.87, 131.94, 130.67, 128.43, 128.37, 128.15, 127.63, 125.79, 125.77, 87.00, 86.97, 56.37, 50.31, 23.75, 19.28

1-(1-(3-nitrophenyl)-3-phenylprop-2-yn-1-yl)pyrrolidine[4i]

Solid; Yield: 83%; ^{1}H NMR ($CDCl_3$, 400 MHz): δ 8.83 (t, J = 1.9Hz, 2H), 8.44 (dd, J = 2.3, 1.1 Hz, 1H), 8.42 (dd, J = 2.3, 1.1 Hz, 1H), 8.36-8.35 (m, 1H), 8.34-8.33 (m, 1H), 7.91-7.87 (m, 2H), ^{13}C NMR ($CDCl_3$, 400 MHz): δ 146.89, 139.58, 134.39, 131.87, 130.03, 129.21, 128.85, 128.82, 128.35, 127.17, 123.37, 120.71, 59.20, 50.36, 23.55.

1-(1-(4-chlorophenyl)-3-phenylprop-2-yn-1-yl)pyrrolidine[4j]

Solid; Yield: 86%; ^{1}H NMR ($CDCl_3$, 400 MHz): δ 7.52-7.47 (m, 6 H), 7.33-7.32 (m, 3H), 4.87 (s, 1H), 2.68 (m, 4H), 1.80 (m, 4H), ^{13}C NMR ($CDCl_3$, 400 MHz): δ 131.88, 129.68, 129.09, 128.82, 128.77, 128.38, 128.36, 127.68, 127.5, 127.51, 86.99, 59.22, 50.37, 23.55.

5. ^{1}H and ^{13}C NMR spectra of synthesised compounds

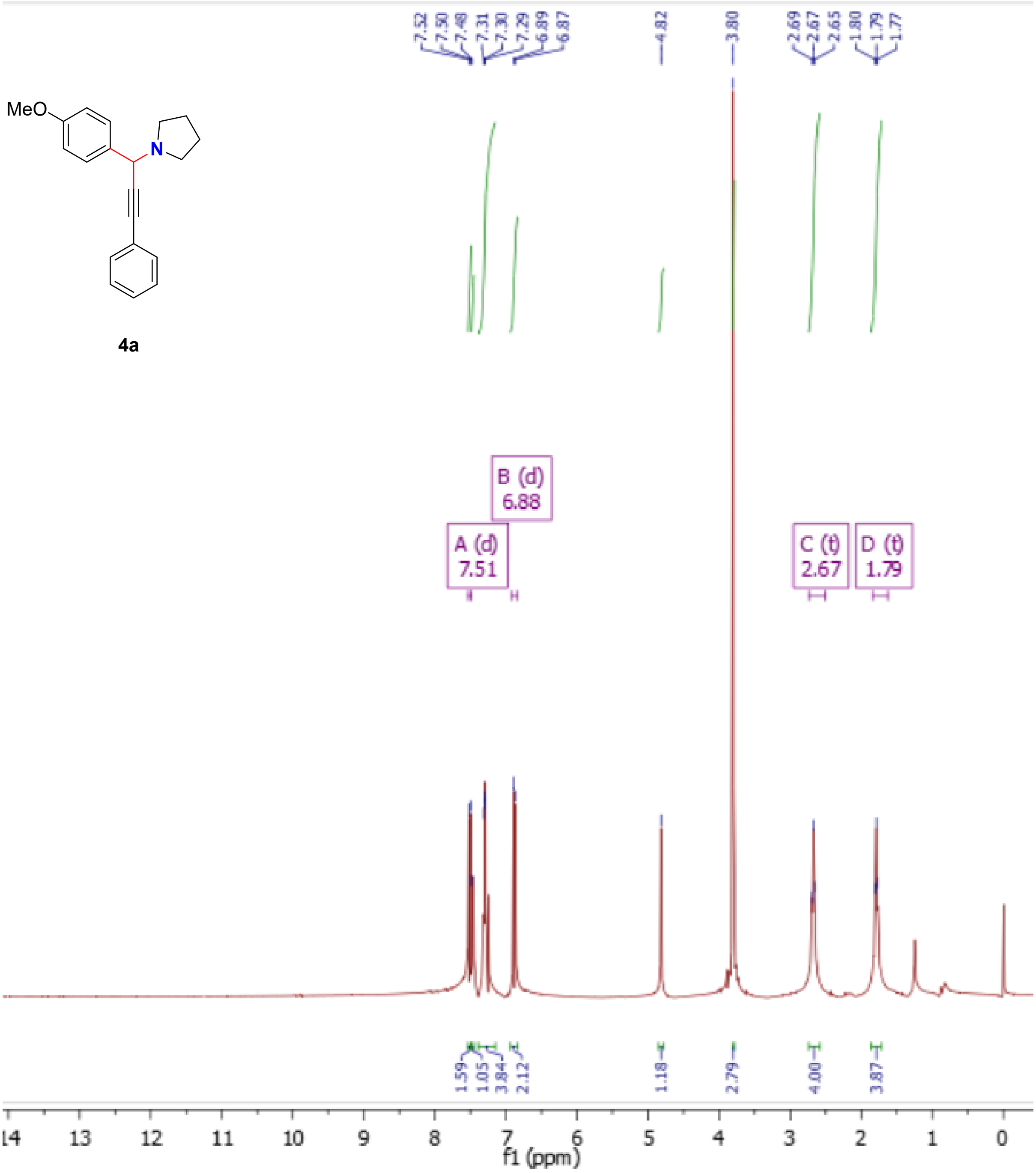

Figure S6: ^{1}H NMR of 1-(1-(4-methoxyphenyl)-3-phenylprop-2-yn-1-yl)pyrrolidine

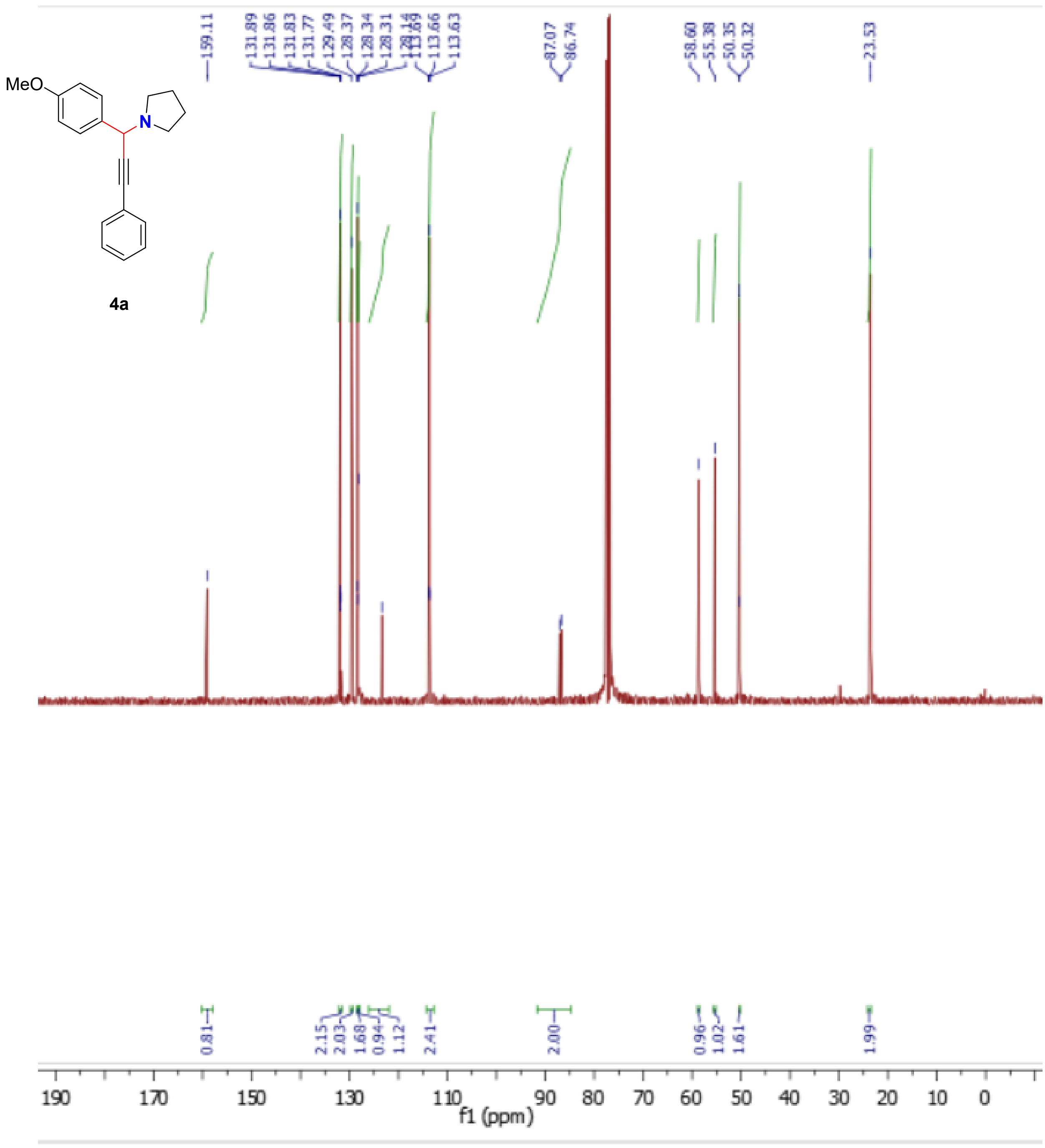

Figure S7: ^{13}C NMR of 1-(1-(4-methoxyphenyl)-3-phenylprop-2-yn-1-yl)pyrrolidine

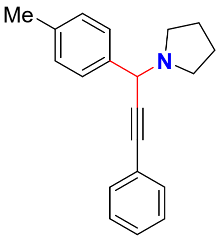

4b

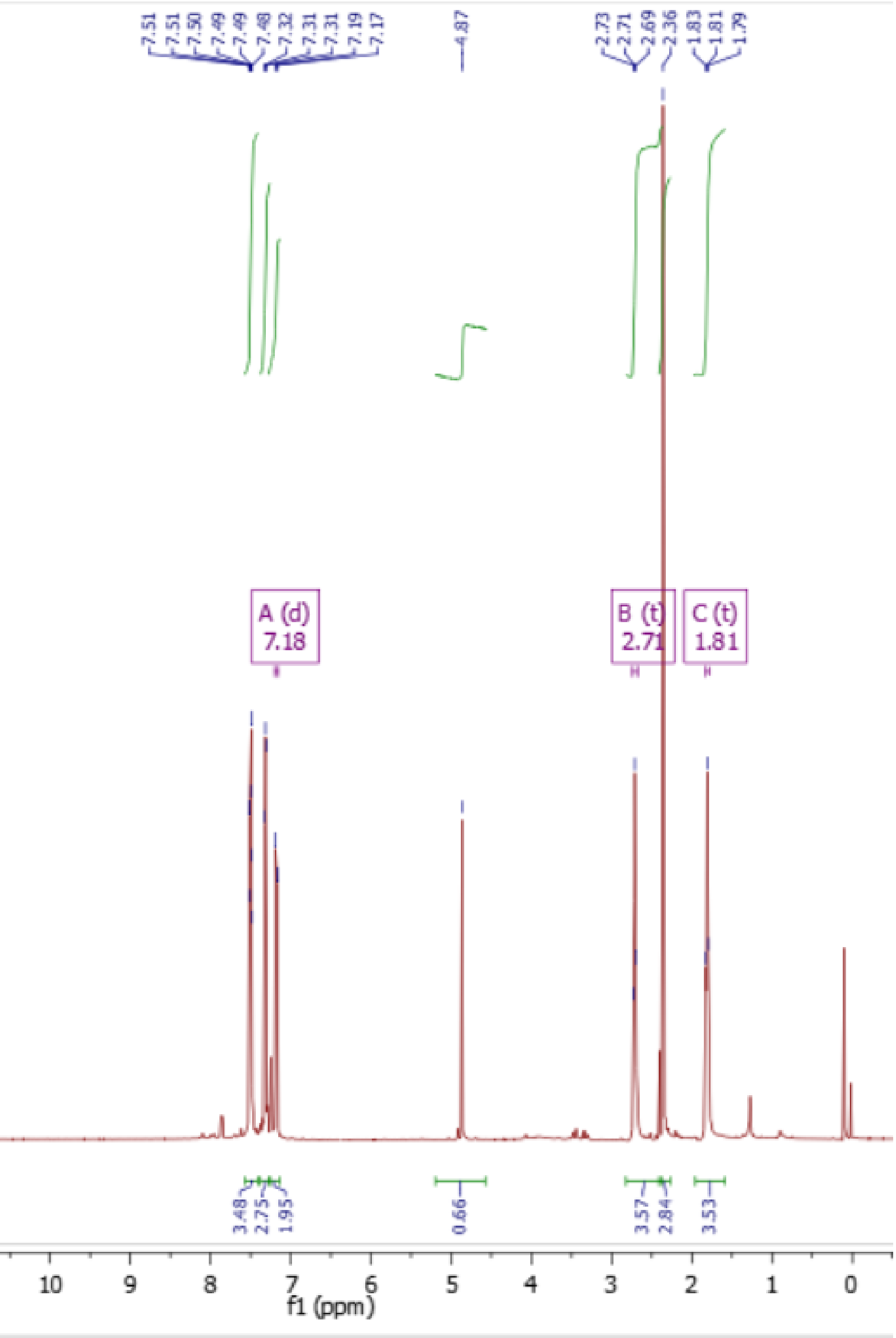

Figure S8: ^{1}H NMR of 1-(3-phenyl-1-(*p*-tolyl) prop-2-yn-1-yl)pyrrolidine

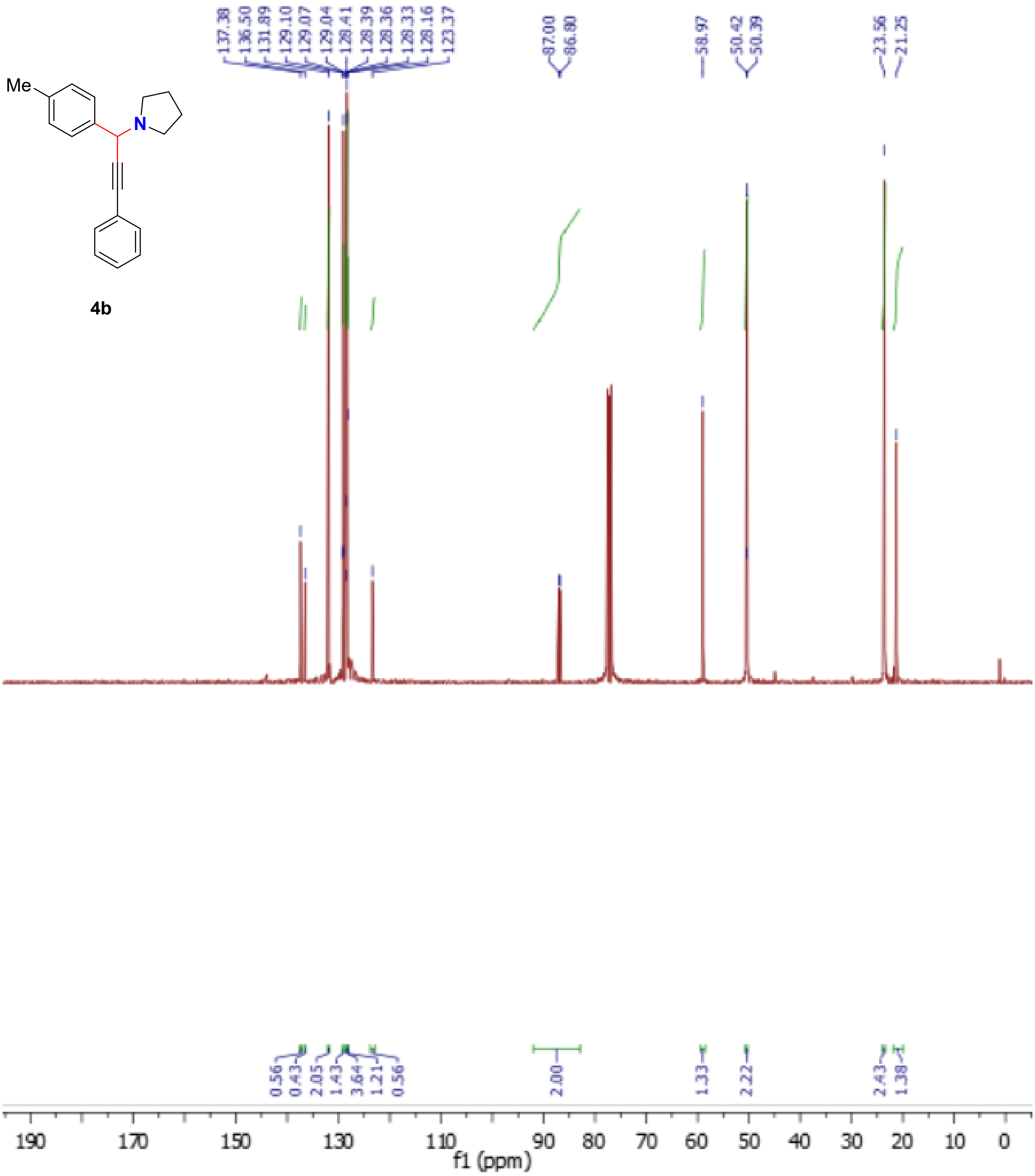

Figure S9: ^{13}C NMR of 1-(3-phenyl-1-(*p*-tolyl) prop-2-yn-1-yl)pyrrolidine

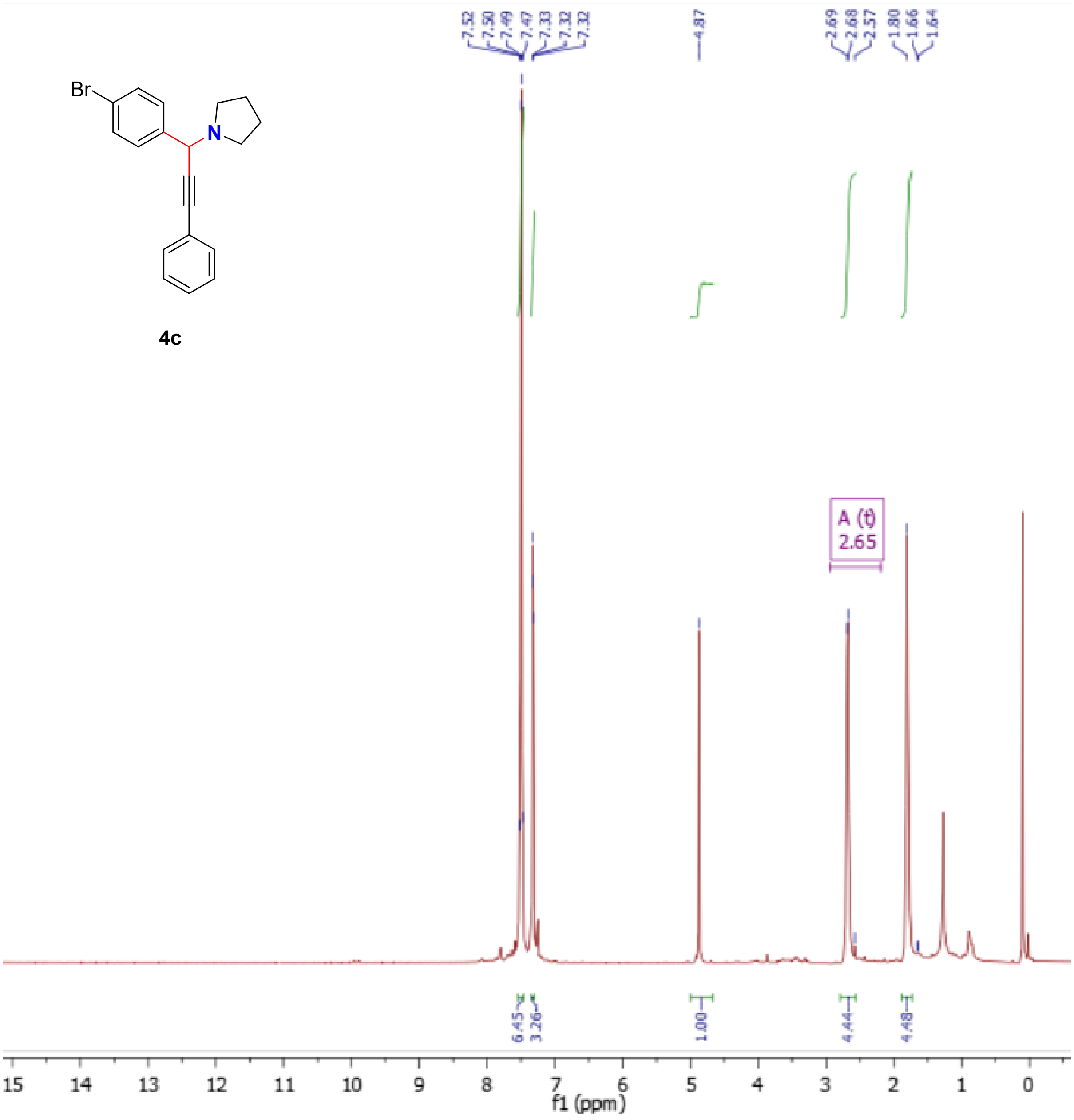

Figure S10: ^{1}H NMR of 1-(1-(4-bromophenyl)-3-phenylprop-2-yn-1-yl)pyrrolidine

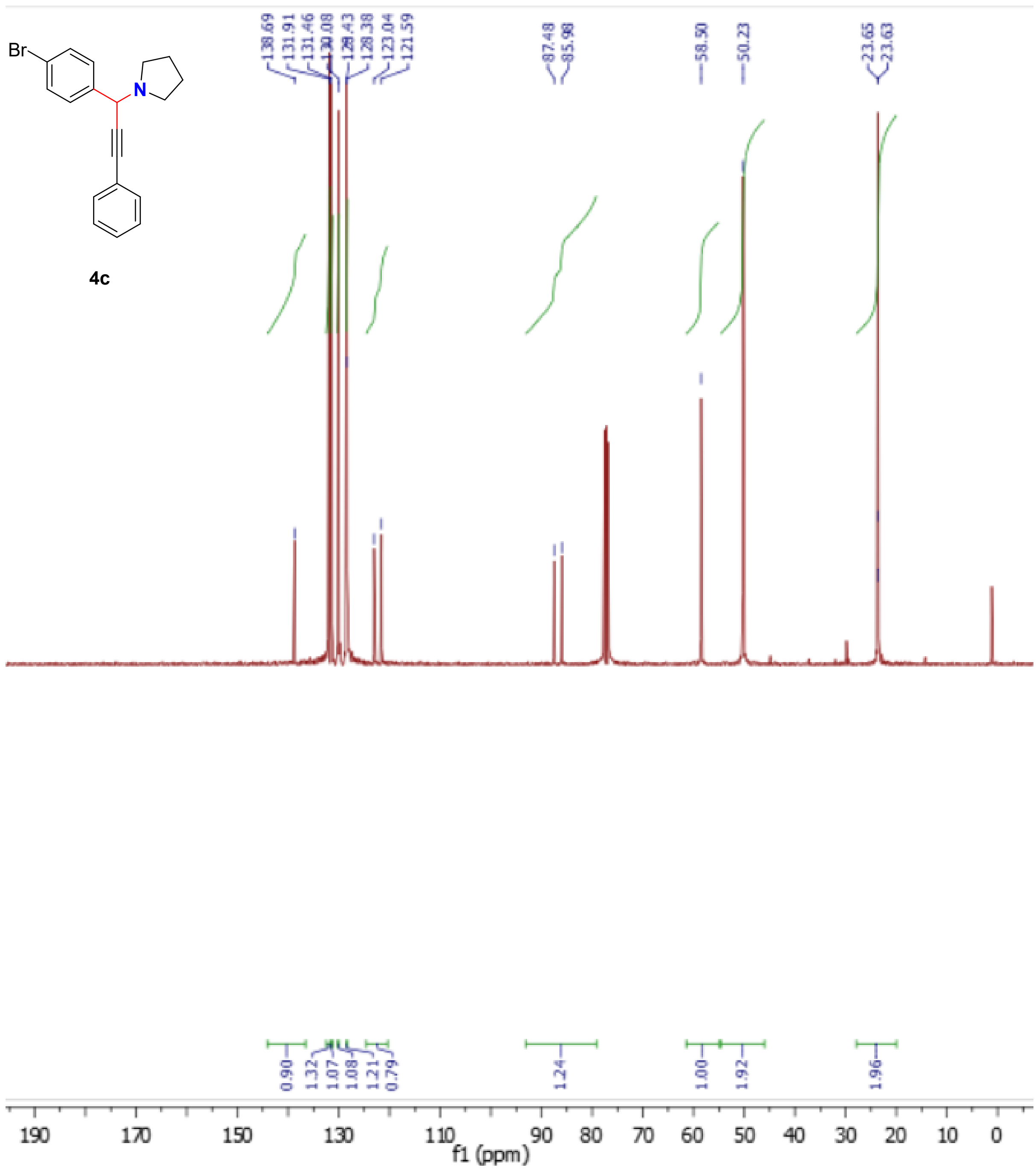

Figure S11: ^{13}C NMR of 1-(1-(4-bromophenyl)-3-phenylprop-2-yn-1-yl)pyrrolidine

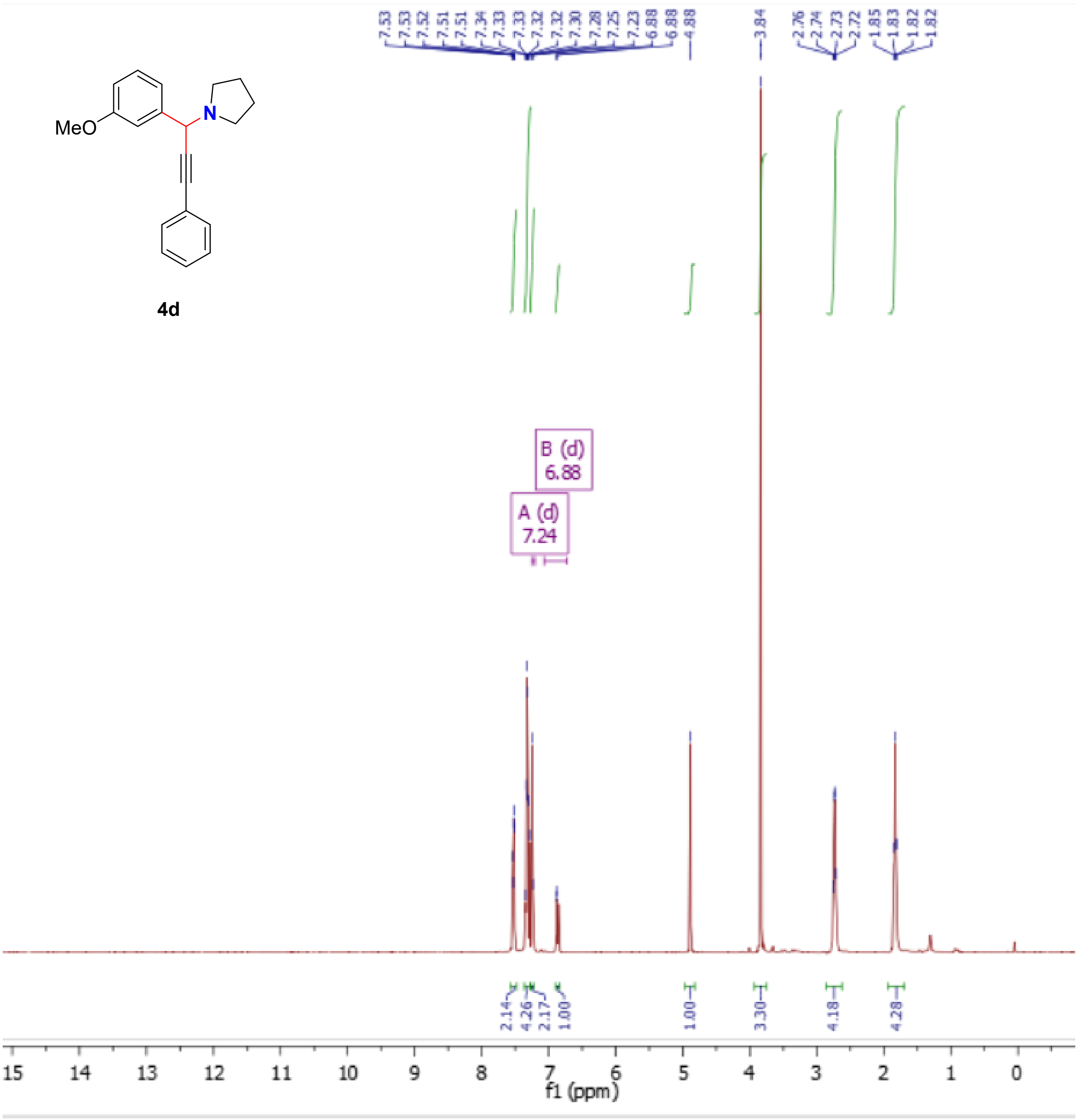

Figure S12: ^{1}H NMR of 1-(1-(3-methoxyphenyl)-3-phenylprop-2-yn-1-yl)pyrrolidine

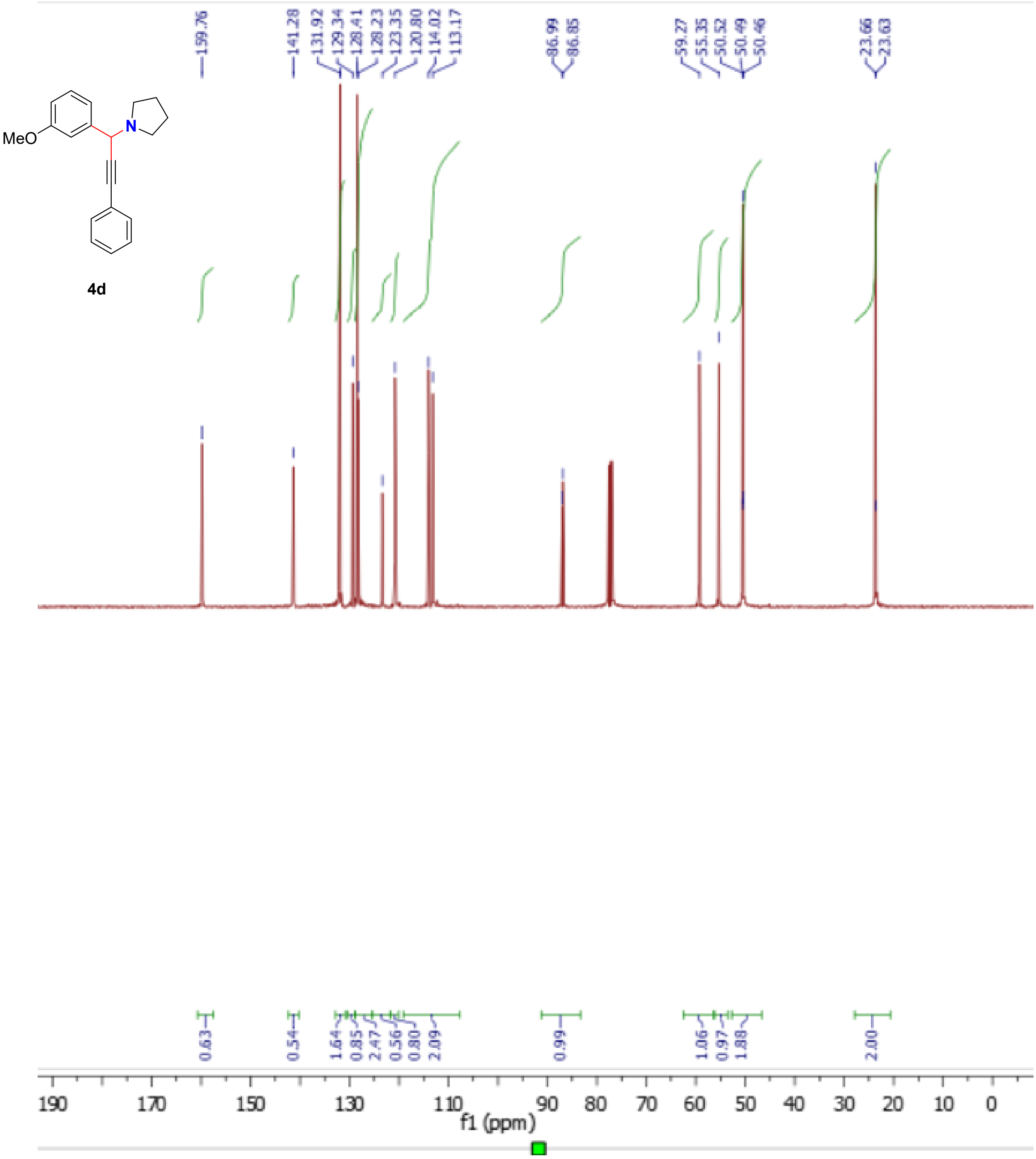

4d

Figure S13: ^{13}C NMR of 1-(1-(3-methoxyphenyl)-3-phenylprop-2-yn-1-yl)pyrrolidine

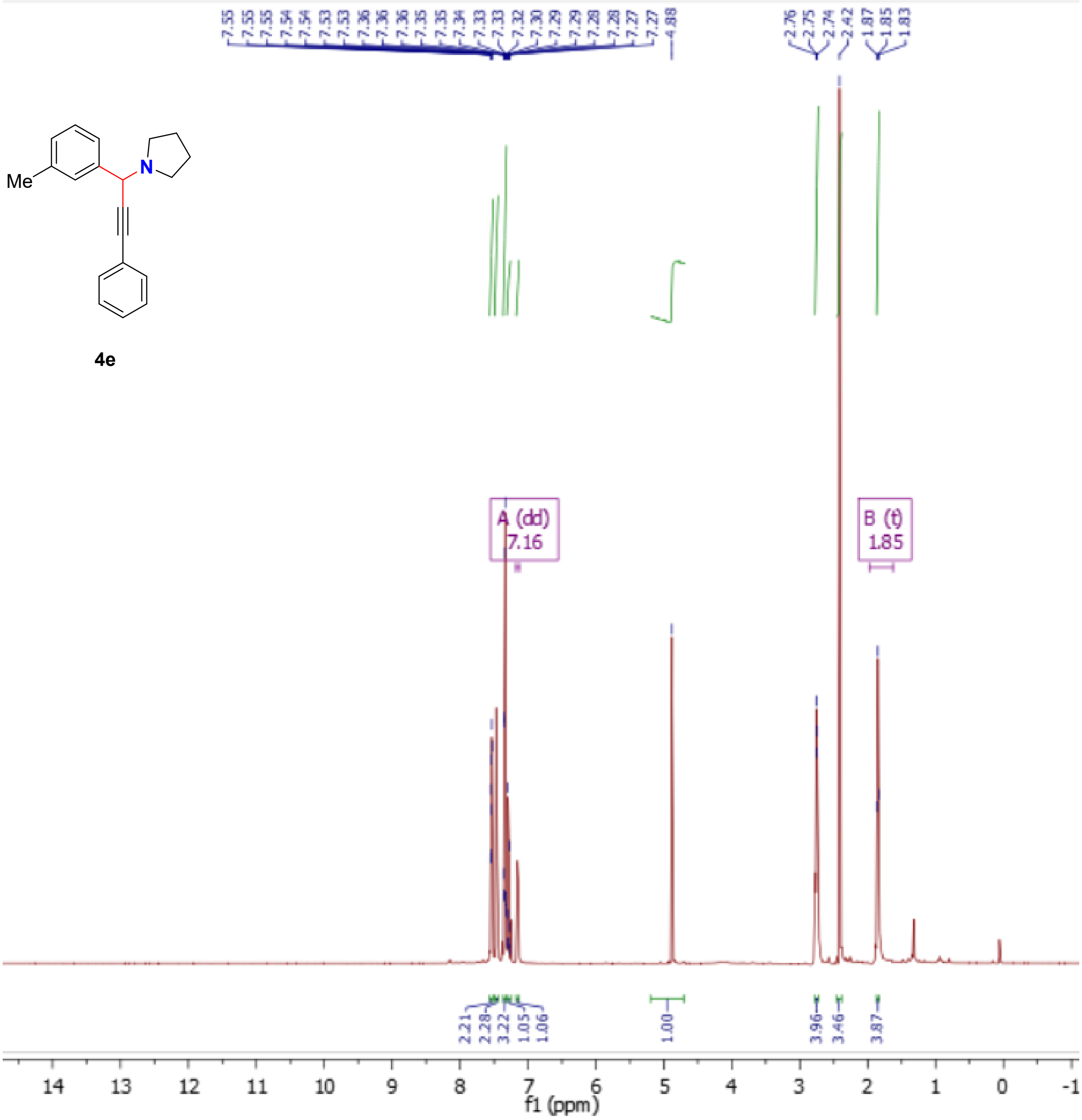

Figure S14: ¹H NMR of 1-(3-phenyl-1-(*m*-tolyl) prop-2-yn-1-yl)pyrrolidine

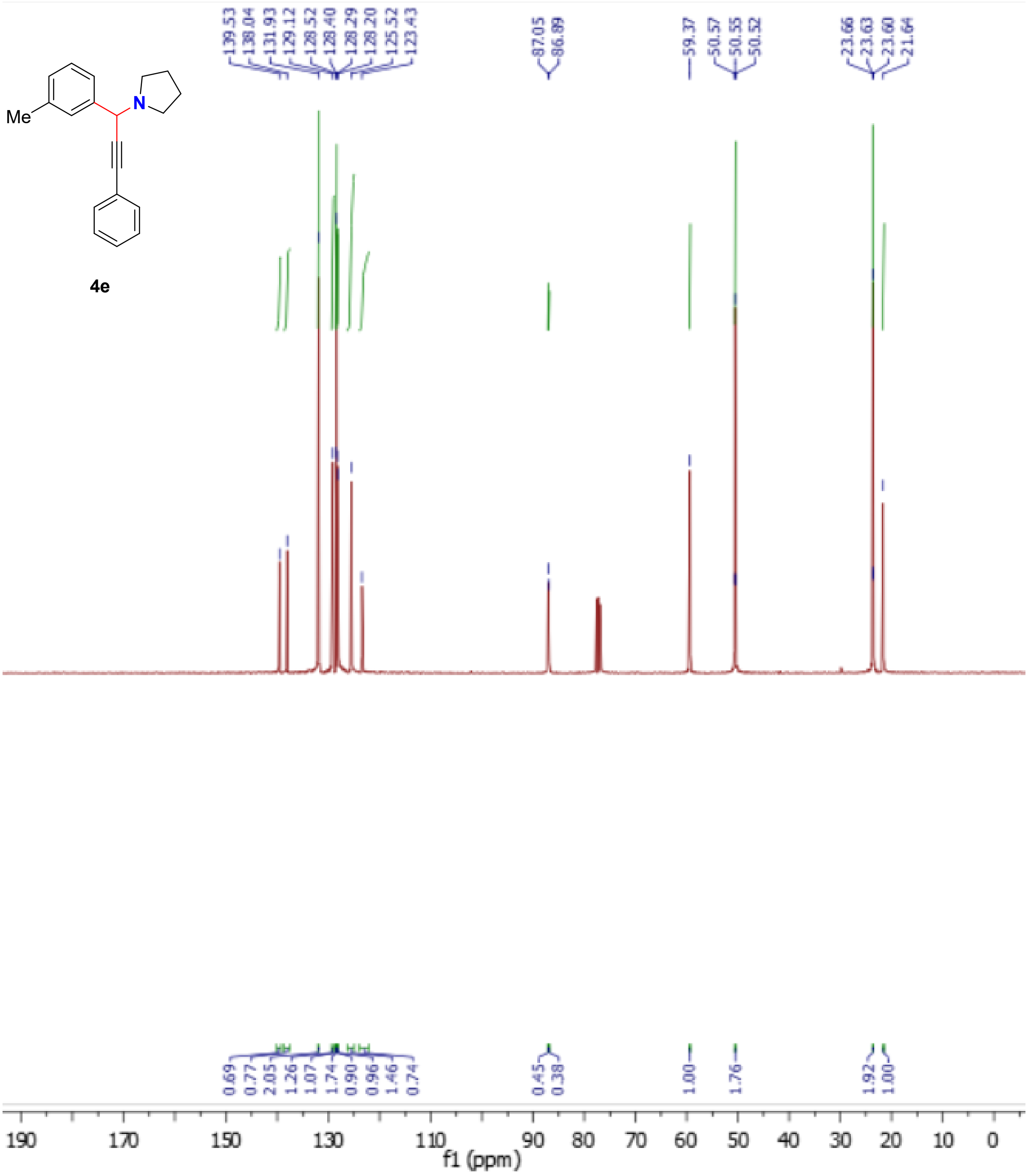

Figure S15: ^{13}C NMR of 1-(3-phenyl-1-(*m*-tolyl) prop-2-yn-1-yl)pyrrolidine

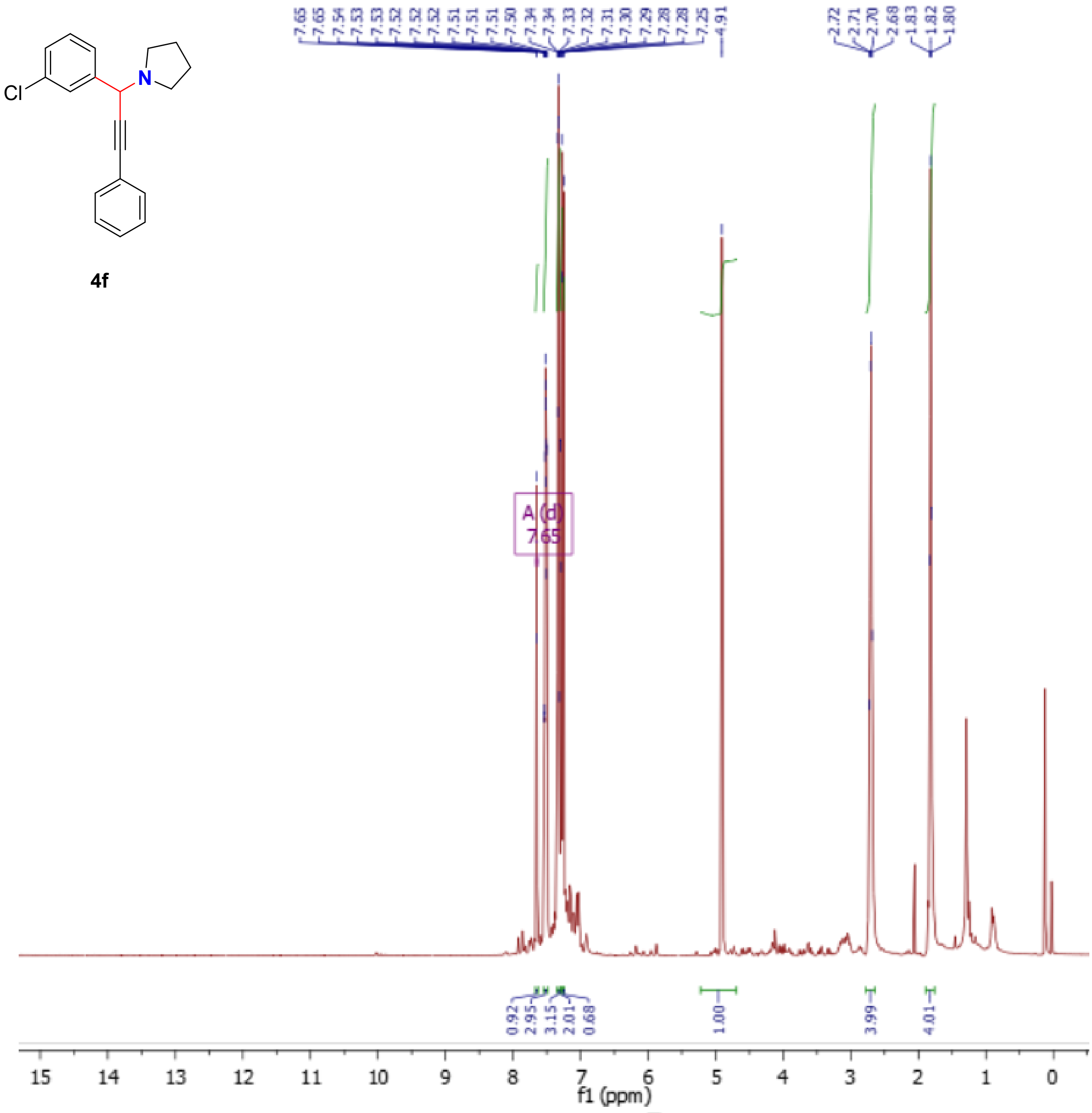

Figure S16: ^{1}H NMR of 1-(1-(3-chlorophenyl)-3-phenylprop-2-yn-1-yl)pyrrolidine

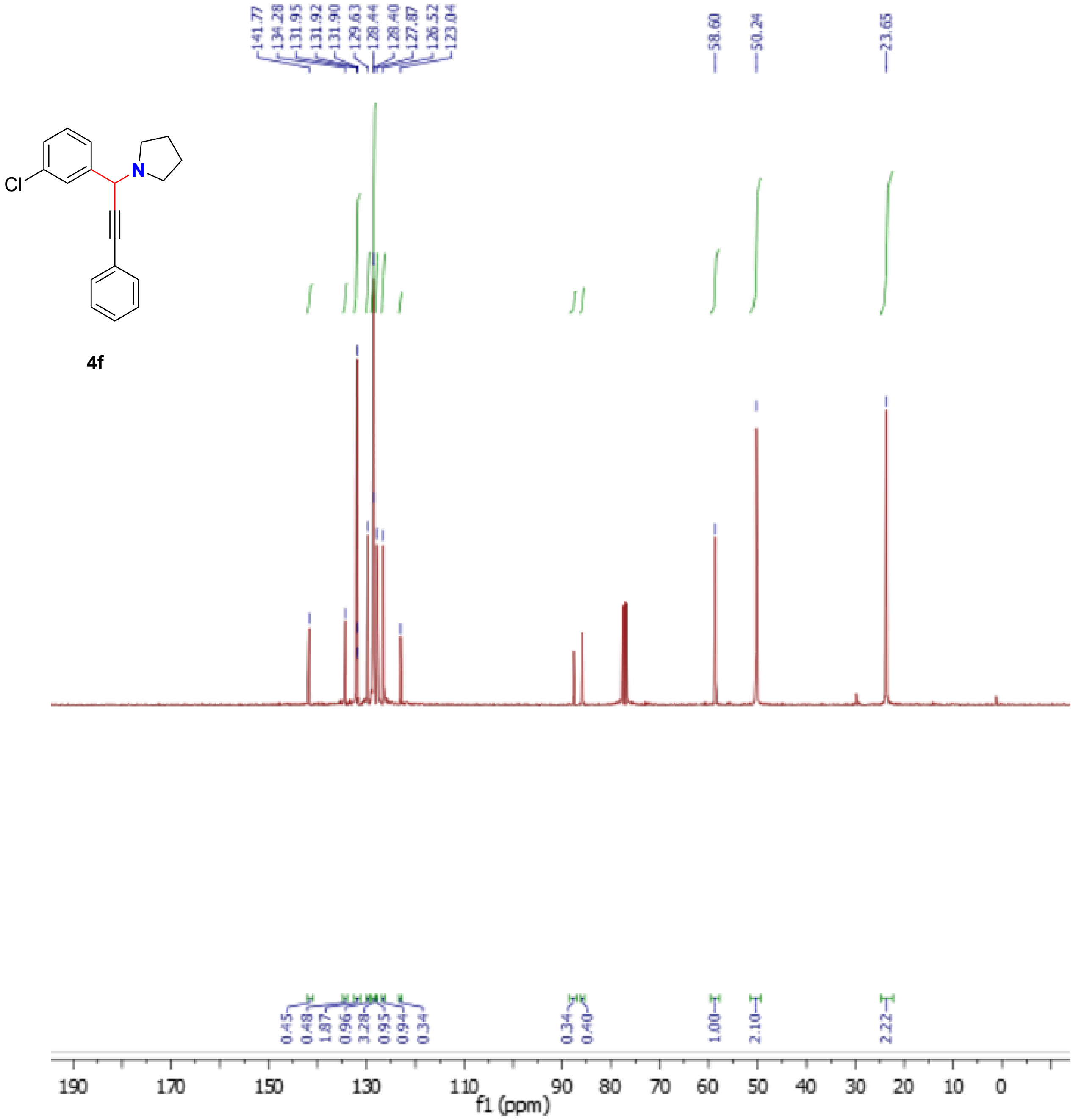

Figure S17: ^{13}C NMR of 1-(1-(3-chlorophenyl)-3-phenylprop-2-yn-1-yl)pyrrolidine

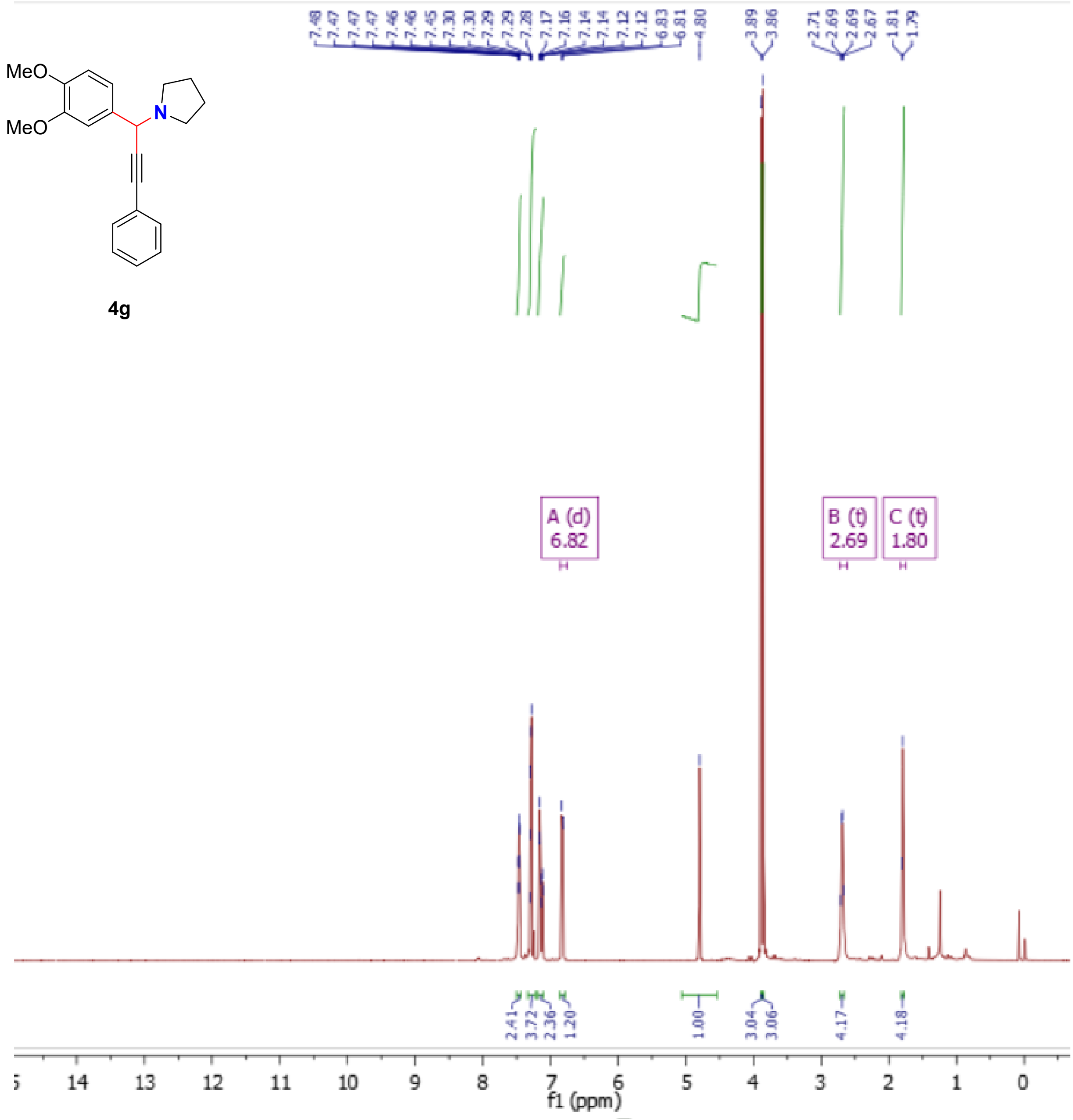

Figure S18: ^{1}H NMR of 1-(1-(3,4-methoxyphenyl)-3-phenylprop-2-yn-1-yl)pyrrolidine

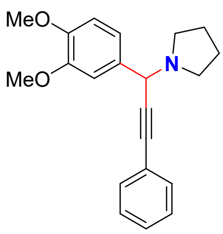

4g

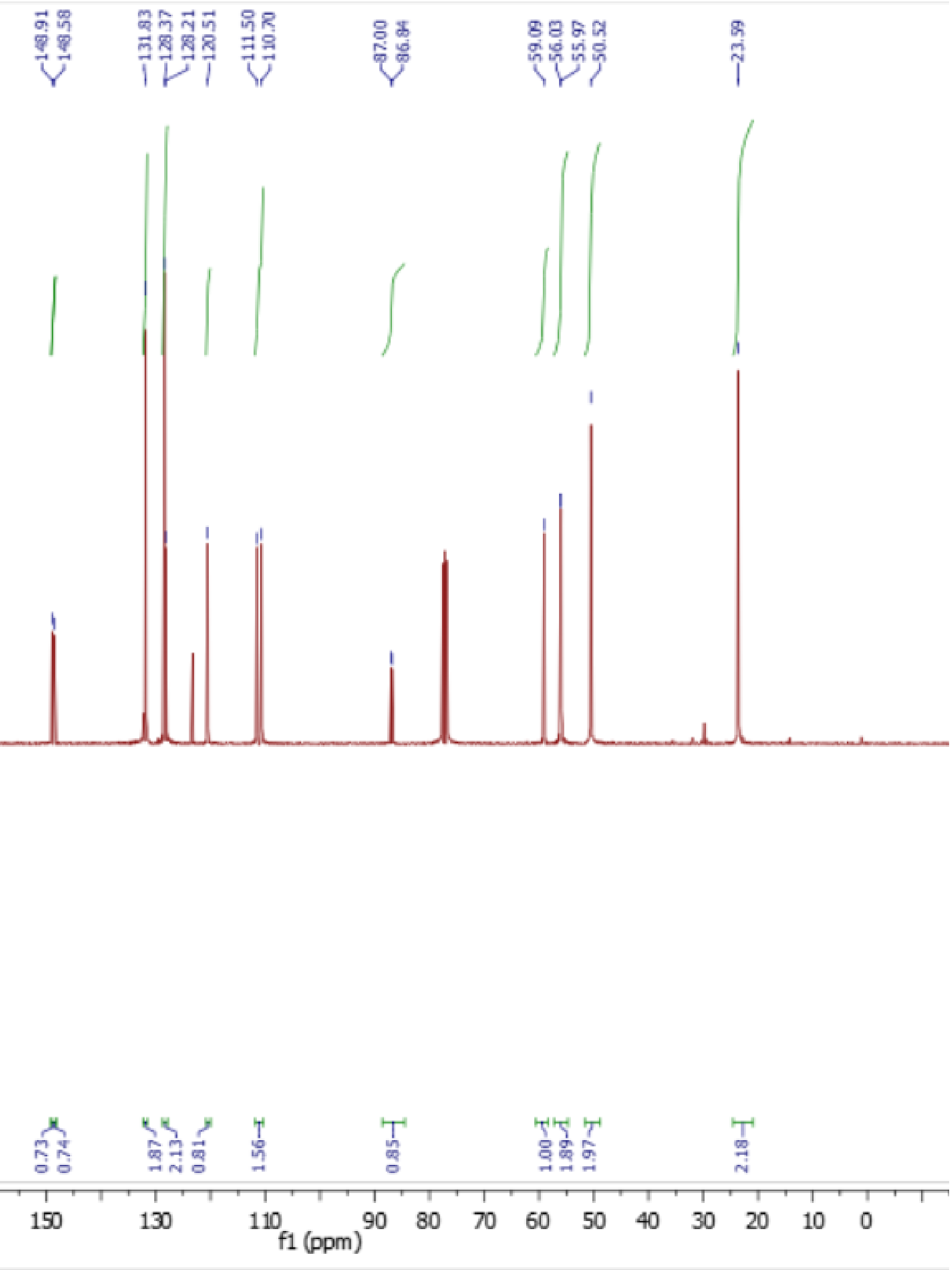

Figure S19: ^{13}C NMR of 1-(1-(3,4-methoxyphenyl)-3-phenylprop-2-yn-1-yl)pyrrolidine

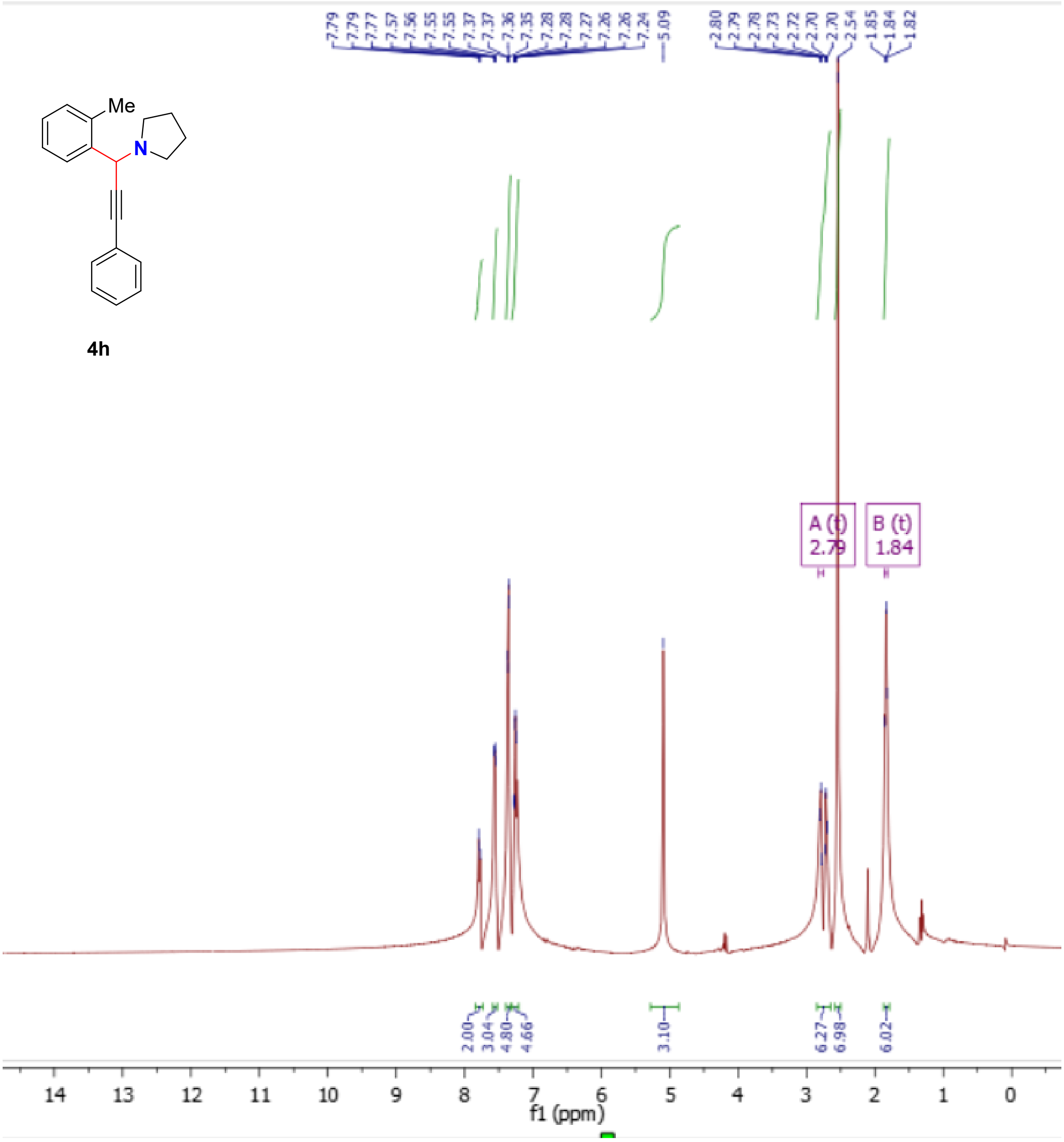

Figure S20: ^{1}H NMR of 1-(3-phenyl-1-(*o*-tolyl) prop-2-yn-1-yl)pyrrolidine

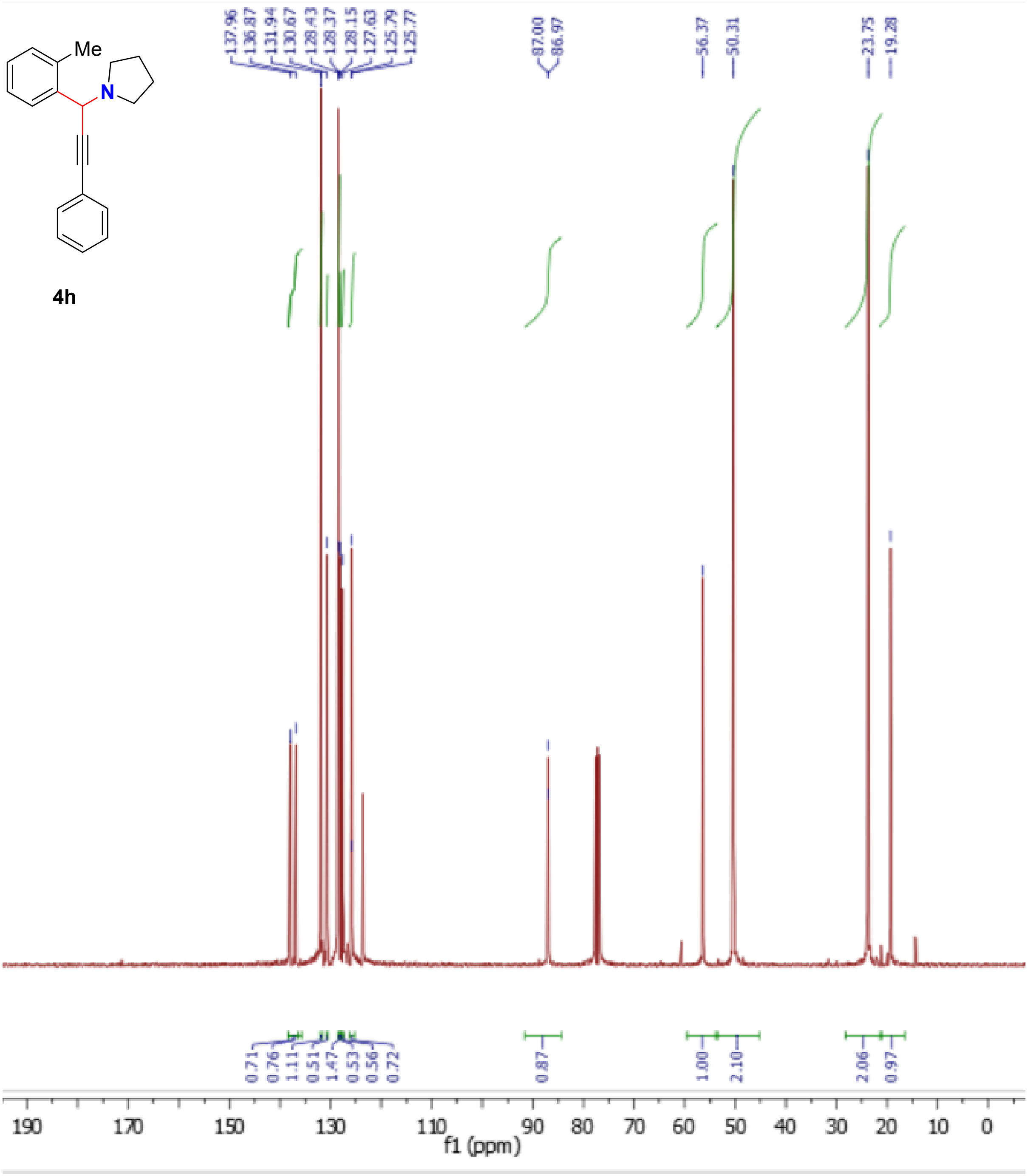

Figure S21: ^{13}C NMR of 1-(3-phenyl-1-(*o*-tolyl) prop-2-yn-1-yl)pyrrolidine

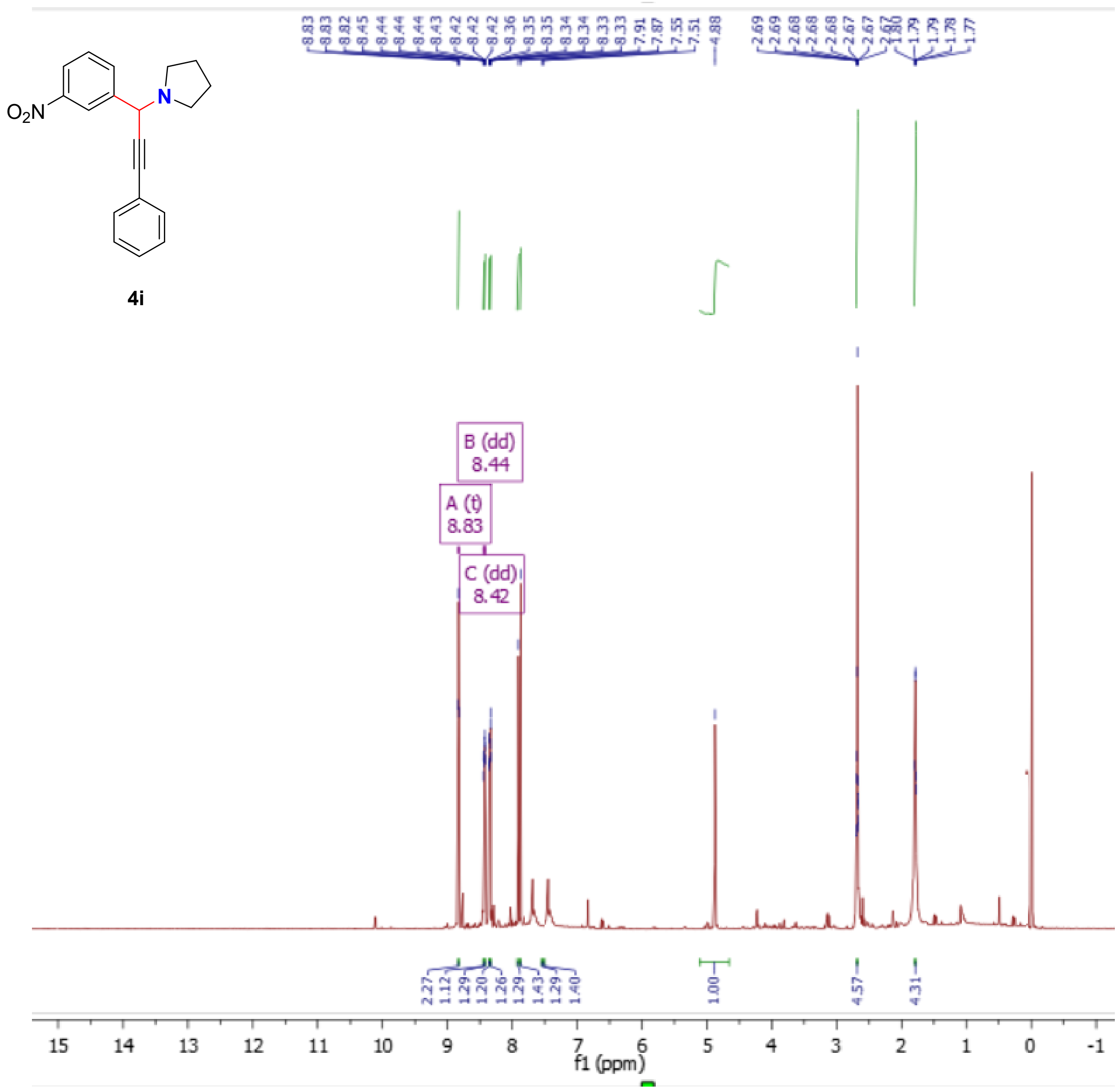

Figure S22: ^{1}H NMR of 1-(1-(3-nitrophenyl)-3-phenylprop-2-yn-1-yl)pyrrolidine

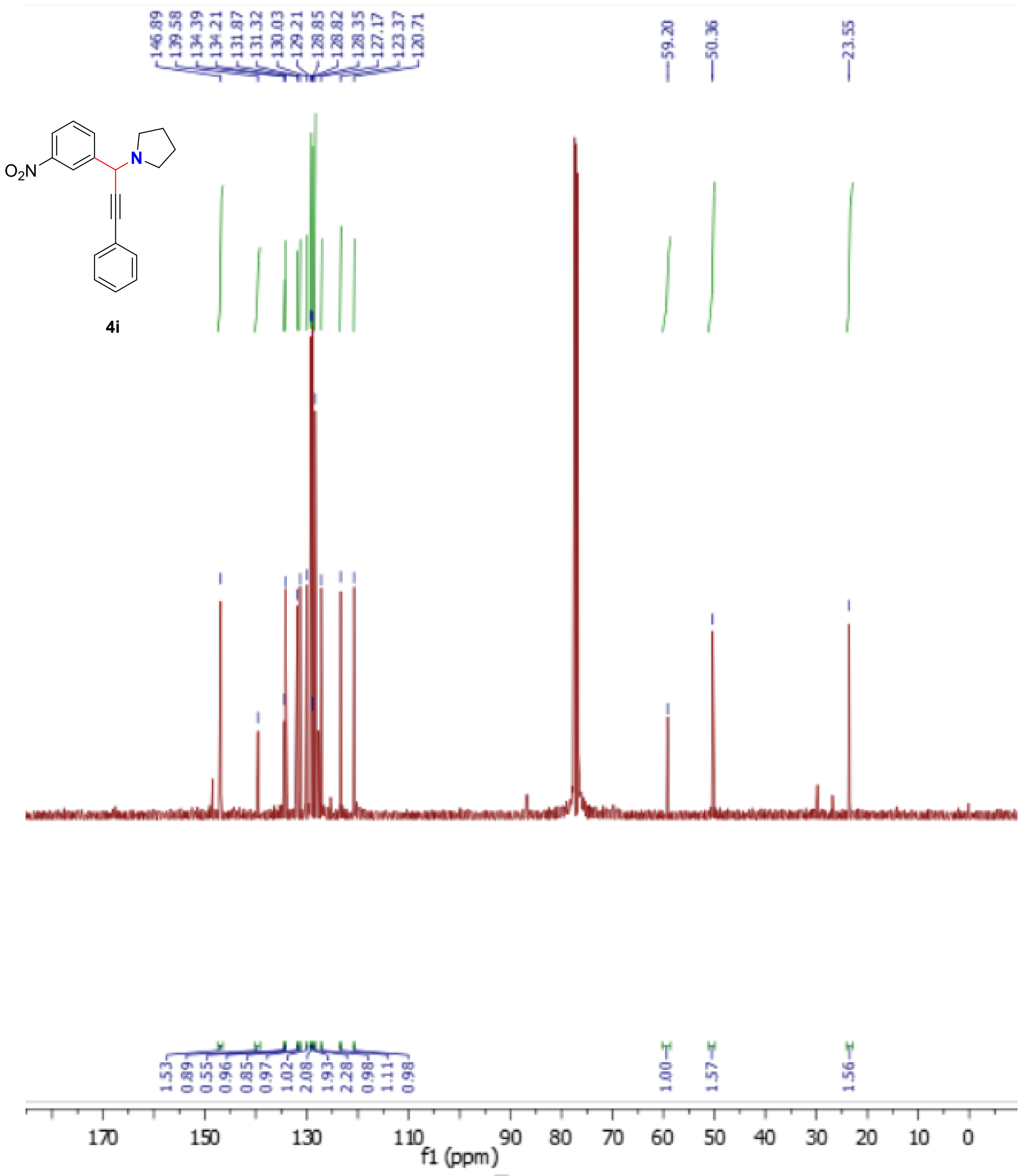

Figure S23: ^{13}C NMR of 1-(1-(3-nitrophenyl)-3-phenylprop-2-yn-1-yl)pyrrolidine

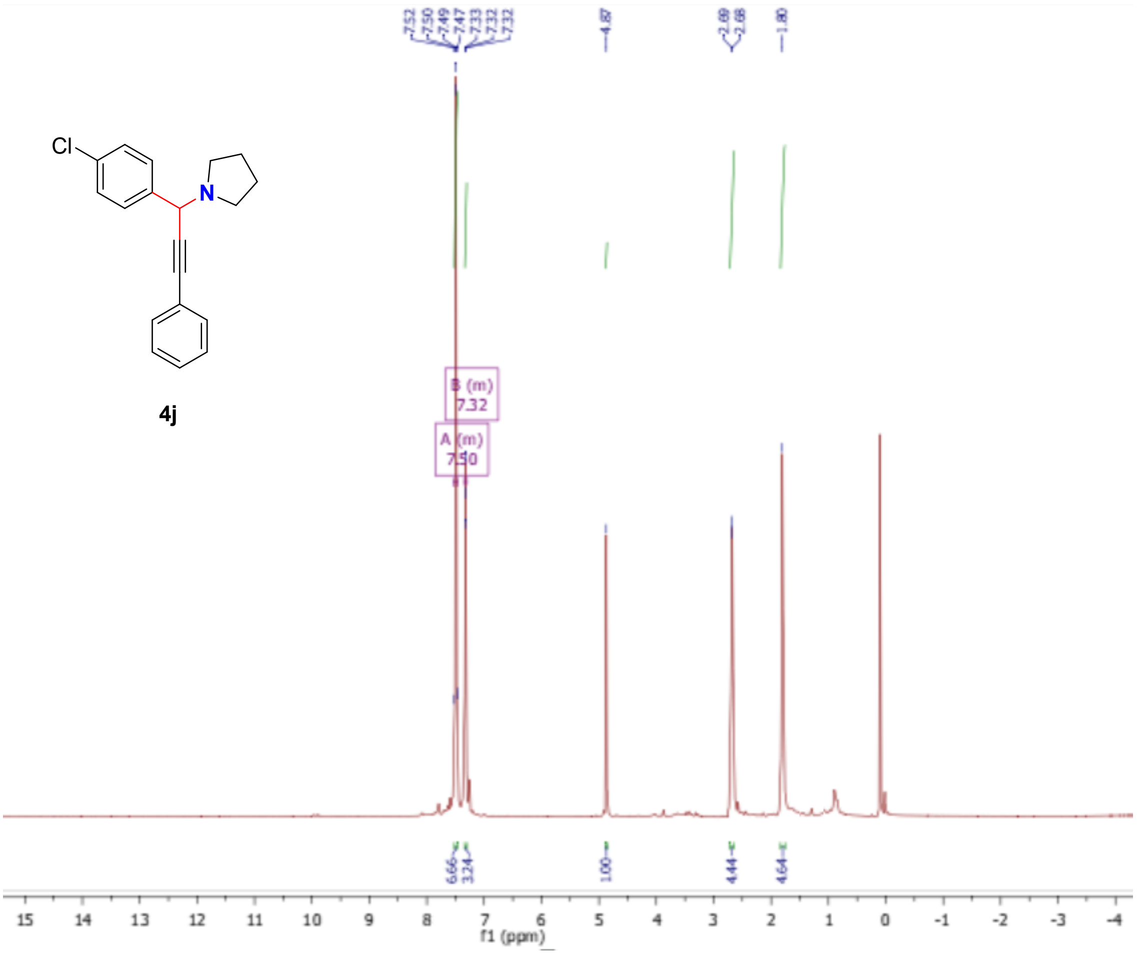

Figure S24: ^{1}H NMR of 1-(1-(4-chlorophenyl)-3-phenylprop-2-yn-1-yl)pyrrolidine

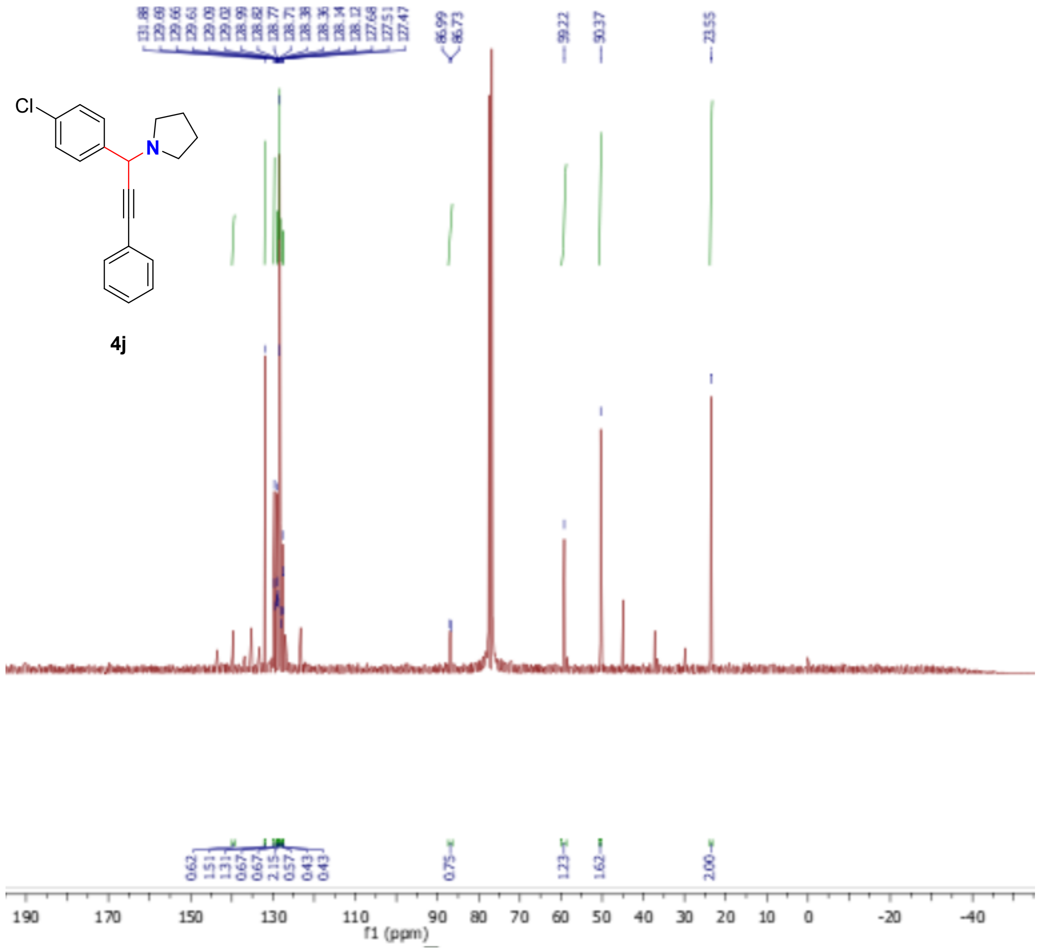

Figure S25: ^{13}C NMR of 1-(1-(4-chlorophenyl)-3-phenylprop-2-yn-1-yl)pyrrolidine
